# Supplementary material for: Proteasome inhibitor-induced modulation reveals the spliceosome as a specific therapeutic vulnerability in multiple myeloma
Source: Nat Commun. 2020 Apr 22;11:1931. doi: 10.1038/s41467-020-15521-4 (PMC7176739; doi:10.1038/s41467-020-15521-4)
Supplement: Supplementary file 1 — Supplementary Information [file 41467_2020_15521_MOESM1_ESM.pdf]

Supplementary Information For:

Proteasome inhibitor-induced modulation reveals the spliceosome as a specific therapeutic vulnerability in multiple myeloma

Hector H. Huang[1], Ian D. Ferguson[1], Alexis M. Thornton[2], Prabhakar Bastola[1], Christine Lam[1], Yu-Hsiu T. Lin[1], Priya Choudhry[1], Margarette C. Mariano[1], Makeba D. Marcoulis[1], Chin Fen Teo[3], Julia Malato[4], Paul J. Phojanakong[4], Thomas G. Martin[5], Jeffrey L. Wolf[5], Sandy W. Wong[5], Nina Shah[5], Byron C. Hann[4], Angela N. Brooks[2], Arun P. Wiita[1,4,*]

[1]Dept. of Laboratory Medicine, University of California, San Francisco, CA [2]Dept. of Biomolecular Engineering, University of California, Santa Cruz, CA, [3]Dept. of Physiology, University of California, San Francisco, [4]Helen Diller Family Comprehensive Cancer Center, University of California, San Francisco, CA, [5]Dept. of Medicine, University of California, San Francisco, CA

This document includes:

Supplementary Methods and References

Supplementary Figures 1 – 11

Supplementary Table 1

The following supplementary files are available as separate .xlsx files:

Supplementary Data 1 - 5 (Excel Files)

Source Data (Excel File)

***Corresponding Author Contact information:**

Arun P. Wiita, MD, PhD

Arun.wiita@ucsf.edu

List of Supplementary Items:

Supplementary Figure 1: Time-course of MM cell phosphorylation after Cfz treatment.

Supplementary Figure 2: Protein abundance and gene expression response to drug perturbation

Supplementary Figure 3: Characterization of myeloma response to Cfz, melphalan, and z-vad-fmk.

Supplementary Figure 4: Cfz-induced splicing alterations across parental MM cells and SRSF constructs.

Supplementary Figure 5: The phosphorylation-dependent interactome of SRSF1.

Supplementary Figure 6: Splicing factor phosphorylation is induced in response to btz and blocked by caspase inhibition.

Supplementary Figure 7: Bortezomib and heat shock induce specific splicing, but not global alternative splicing in MM.1S.

Supplementary Figure 8: Cfz induces SRSF1 localization in the nuclear periphery of AMO-1.

Supplementary Figure 9: E7107 cell toxicity, functional splicing assay, and splicing statistics.

Supplementary Figure 10: E7107 has specific anti-MM activity in synergy with PI.

Supplementary Figure 11: Preclinical and clinical relevance of targeting the spliceosome in myeloma.

Supplementary Table 1 Description of SRSF1 lentiviral expression constructs.

Supplementary Data 1: Excel file with tabs listing comparative analysis (T-test *p*-value and $\log_2$-difference) of phospho- and global proteomics

Supplementary Data 2: Oligo sequences for cloning, qPCR, and RNA-seq cDNA library.

Supplementary Data 3: Distribution statistics of ΔPSI calculated in R. Statistics for individual event types and all event types are listed for each comparative condition.

Supplementary Data 4: Excel file with tabs summarizing bioinformatics GO enrichment analysis and KSEA kinase scores.

Supplementary Data 5: Excel file listing splicing gene mutations and variant allele frequencies found in MM patients from CoMMpass dataset.

Source Data: excel file with uncropped immunoblot images shown in Fig 6e, Supplementary Fig. 3c-3g, and Supplementary Fig. 4e.

Supplementary Methods

Cell lines

MM.1S is from ATCC (CRL-2974); AMO-1 and AMO-1 Btz-resistant are gifts courtesy of Dr. Christoph Driesen (Kantonsspital St Gallen); L363, RPMI8266, JJN3 are from Deutsche Sammlung von Mikroorganismen und Zellkulturenrepository (DSMZ; ACC 49, ACC 402, ACC 541 respectively); INA6 is a gift courtesy of Dr. Renate Burger (Christian-Albrechts-Universität zu Kiel); KMS34 is from Japanese Collection of Research Bioresources Cell Bank (JCRB1195); and MM.1S mCherry/f-luc is a gift of Dr. Diego Acosta-Alvear, UCSF. All cell lines used are female, except INA6 and RPMI8266. All cell lines were validated using STR profiling service by ATCC.

Cell culture for PI-response proteomics

For single-timepoint experiments, cells were passaged in either light SILAC media (SILAC RPMI 1640 (Thermo, PI88421), supplemented with 1% Pencillin/Streptomycin, 10% dialyzed FBS (Thermo, PI88440), and 321.6 µM L-Lysine and 190.4 µM L-Arginine (Sigma, L8662-25G, A6969-25G) or heavy SILAC media (L-Lysine-$^{13}C_6$,$^{15}N_2$, L-Arginine-$^{13}C_6$,$^{15}N_4$ (Cambridge Isotope, CNLM-291-H-1, CNLM-539-H-1) instead of L-Lys and L-Arg) for more than 6 doublings previous to drug dosing experiments to mostly incorporate heavy and light lysine and arginine. For SRSF1 AP-MS, ~30E+6 cells were treated in complete media with 15 nM Cfz or DMSO for 24 hr. Cells were harvested by centrifugation at 300 rcf for 5 min and washed with 5 mL PBS. Cells were then pelleted by centrifugation, PBS was aspirated, and cell pellets were frozen in liquid nitrogen (LN2) and stored in -80°C.

E7107 timecourse and washout

For E7107 time-course “washout” experiment in **Supplementary Fig. 9a**, cells are treated in 6-well plates at 1E+6 cells/mL with DMSO, 0.1 nM, 2 nM E7107 for AMO-1 and DMSO, 4 nM , and 10 nM E7107 for MM.1S . Cell viability was measured with CellTiter-Glo (Promega, G7573) at 0 hr, 6 hr, 24 hr, and 48 hr, where media for “washout” samples were exchanged for fresh media, without E7107after the 6 hr measurement.

SRSF1 Cloning details

Refer to Supplementary Data 2 for primer sequences and **Supplementary Table 1 and Supplementary Fig. 4c** for construct details and assembly of lentiviral vectors encoding SRSF1-mCherry-(NLS)-$[FLAG]_3$ (with and without NLS) and its RS domain mutants used in SRSF1 AP-MS experiments, microscopy experiments, and AMO-1 exogenous SRSF1 alternative splicing analysis.

Plasmid encoding SRSF1 was isolated from Human ORFeome library v8.1 (1) (Access provided by UCSF Recombinant Antibody Network), and polymerase chain reaction (PCR) was used to amplify the gene from this plasmid, nucleoplasmin nuclear localization signal (NLS) and 3X FLAG sequences from PX458, and pLV lentiviral transfer plasmid backbone and mCherry from pLV-416G. Gibson assembly was used to combine SRSF1, mCherry, NLS, and 3X FLAG with the lentiviral vector backbone (excluding luciferase-T2A-mCherry genes) pLV-416G and this WT construct is referred to as A4. SRSF1 mutant constructs were formed by PCR amplification of a truncated SRSF1 (1-197) in A4, and Gibson assembly with gBlock oligos (IDT), synthetically designed oligos substituting native codons for all Ser with codons for Asp or Ala, depending on it being a SD or SA mutant. SD construct is referred to as I1, while SA construct is referred to as III1. However, SA gBlock design left a shortened linker region. This was corrected by further PCR amplification of III1 plasmid with extended primers to create corrected SA referred to as X3. AP-MS negative control of mCherry-(NLS)-$[FLAG]_3$, referred to as ctrl1, was constructed by PCR amplification of A4 with primers excluding SRSF1 and annealed by one fragment Gibson Assembly. Template vector was removed by DpnI nuclease treatment.

Lentiviral transduction details

For each sample, 1.5 µg lentiviral-SRSF1 transfer plasmid was transfected along with 1.33 µg of the packaging plasmid pCMV-dR8.91 (containing Gag-Pol) and 0.17 µg of the VSV-G envelope expressing plasmid pMD2.G into Lenti-X (Takara, 632180) packaging cells (seeded the day before in 6-well plates with 0.6E+6 cells and 2.6 mL

Opti-MEM (Life Tech, 31985-062) per well) with FuGene (Promega, E2311) transfection reagent in 300 µl Opti-MEM, incubating for 30min, before adding to each well. After 2 days transfection, viral particles were harvested and filtered with 0.45 µm filter and concentrated with 1 part viral titer and 3 part Lenti-X concentrator (Takara, 631231, ~9-10 mL total) by incubating at 4°C for more than 12 hr, then spinning at 1500 rcf for 45 min at 4°C. Supernatant is carefully aspirated and virus is resuspended in PBS. Entire viral titers were distributed between AMO-1 and MM.1S cells. ~0.75-1.5E+6 cells were seeded in each well of a 6-well plate with 1.5 mL normal growth media, with 8 µg/mL polybrene added (final concentration of 4 µg/mL) and 1mL of virus and 0.5 mL media, then mixed together. Cells were transduced by spinfection, spinning plates at 1000 rcf at 33°C for 2 hr. Afterwards, plates are stored in 37°C incubator (5% CO_2) for 2 days, before media is replaced. After a few passages, positively transduced cells with G418 (VWR, 970 3-058), for several passages and then sorted for mCherry fluorescence by Fluorescence Activated Cell Sorting (FACS, Sony SH800). Cells were maintained as all other MM cell lines.

LC-MS/MS settings

All samples were analyzed by means of a 3h 15 min non-linear gradient from 2.4% acetonitrile (ACN), 0.1% FA to 32% ACN, 0.1% FA, at 0.2 µL/min, 6 min linear gradient to 79% ACN, 0.1% FA at 0.5 µL/min, then washed with flowrate 0.5 µL/min at 79% ACN, 0.1% FA, for 7 min, except AP-MS peptides, which were analyzed with a 1h 23 min linear gradient from 2.4% ACN, 0.1% FA to 32% ACN, 0.1% FA, at 0.2 µL/min, 2 min linear gradient to 79% ACN, 0.1% FA, ramping flowrate from 0.3ul/min to 0.4 µL/min, then washed at 79% ACN, 0.1% FA, for 5 min ramping from 0.4 to 0.5 µL/min. For label free phosphoproteomics, SILAC global proteomics, and AP-MS, MS1 scan range is from 350 to 1500 m/z, at resolution 70,000, with Top 15 ions (Top 12 for timecourse) selected for MS2 sequencing at resolution 17,500, normalized collision energy (NCE)=27 after each survey scan. For SILAC phosphoproteomics, MS1 scan range is from 300 to 1750 m/z, at resolution 70,000. Top 12 ions are selected for MS2 sequencing at resolution 35,000, NCE=28 after each survey scan. All MS2 isolation windows are 1.7 m/z with 20 s of dynamic exclusion.

Maxquant analysis

Initial timecourse unlabeled phosphoproteomics data were processed together on Maxquant v1.5.1.2 with the following settings: Fixed modifications = "Carbamidomethyl (C), Variable modifications = "Oxidation (M)," "Acetyl (Protein N-term)," and "Phospho (STY)," PSM/Protein FDR = 0.01, min. peptide length = 7, matching time window for matching between runs = 2 min, with 20 min alignment time and all other default parameters (2). Phosphopeptides were searched against the human proteome (Uniprot downloaded 2014/12/3, with 89,706 entries). All SILAC samples were processed together on Maxquant v1.6.0.16 with the same settings, except min. peptide length = 6, matching time window alignment time = 15 min, and max. missed cleavages = 9 (since RS domains on splice factors contain many repeating arginines). Proteomics and phosphoproteomics were searched against the human proteome (Uniprot downloaded on 2018/3/2, with 93,786 entries). SILAC quantification for global proteomics at the protein level requires 1 minimum razor or unique peptide and uses all unmodified and "Oxidation (M)" and "Acetyl (Protein N-term)" modified peptides. AP-MS samples were also processed together on Maxquant v1.6.2.1with the same settings except Variable modifications = "Oxidation (M)," "Acetyl (Protein N-term)," matching time window alignment time = 20 min. Proteins were searched against the human proteome (Uniprot downloaded on 2017/11/15, with 71,544 entries).

Proteomic quantifications, except for the timecourse study, were further evaluated in Perseus (v. 1.6.2.2), where potential contaminants, reverse dummy sequences, and proteins identified by site alone for protein level quantification are excluded (3). Phosphosites with localization scores < 0.75 were also excluded from analysis. Gene ontology annotations were included to identify splicing related proteins. Two biological replicates were grouped and entries with less than 2 valid quantifications were filtered from the final analysis.

Kinase Substrate enrichment analysis

Entire phosphoproteomic results for MM.1S treated with 18 nM Cfz and MM.1S treated with 10 µM melphalan (see **Supplementary Data 1**) were submitted to kinase substrate enrichment analysis accessed through KSEAapp R package (https://github.com/casecpb/KSEAapp/) (4). Gene name and phosphosite, along with fold change and associated T-test p-value statistic were input to generate kinase activity scores, listed in **Supplementary Data 4**. Bar graphs in **Figure 2f** show top ranked kinases with at least 5 substrates (kinases with 4 or less were excluded from graph).

Stress Response Immunoblot

Investigating stress response in **Supplementary Fig. 3**, 1E+6 cells/mL of AMO-1 and MM.1S were treated with DMSO or specified amounts of Cfz or melphalan for 24 hr, harvested, washed with PBS and pelleted by centrifugation at 300 rcf for 5 min, and the cell pellets frozen in 5E+6 cell aliquots in LN2 then stored in -80°C. Cells are thawed and lysed in 125-250 µl 1X RIPA buffer (Millipore) with 1X HALT protease/phosphatase inhibitor (Pierce, 78442), and sonicated with 3 bursts of 5 seconds ON, 10 seconds OFF, @ 20% amplitude with a tip sonicator (BRANSONIC). Protein concentration was quantified with either BCA protein assay kit (Pierce, 23225) or 660 nm protein assay reagent (Pierce, 22660) and ~25-40 µg lysate is loaded per lane and kept consistent across all lanes. Lysate is separated by SDS-PAGE and transferred to PVDF membrane (EMD Millipore, IPFL00010). Membrane is stained with Ponceau-S to assess consistent protein load, then blocked with 5% BSA (Millipore-Sigma, 2930-100GM) in TBS-Tween (0.1% Tween 20 (Fisher Scientific BP337500), TBS (Teknova T1680)) buffer. All primary antibodies are diluted 1:1000 v/v, while secondary antibodies are diluted 1:3000 v/v in 5% BSA in TBS-Tween buffer, unless otherwise specified. Primary antibodies for probing stress response initiation are anti-PERK [clone D11A8] (CST, 5683P; RRID:AB_10841299), anti-BiP (CST, 3183S; RRID:AB_10695864), anti-phospho-eIF2α Ser51 [clone D9G8] (CST, 3398P; RRID:AB_2096481), anti-eIF2α [clone D7D3] (CST, 5324S; RRID:AB_10692650), anti-phospho-4EBP1 Thr37/46 [clone 236B4] (CST, 2855S; RRID:AB_560835), and anti-4EBP1 [clone 53H11] (CST, 9644P; RRID:AB_2097841). Horseradish Peroxidase conjugated antibodies: anti-beta-actin [clone 13E5]-HRP (CST, 5125S; RRID:AB_1903890), secondary anti-Rabbit F(ab)-HRP

(Southern Biotech, 4052-05) and secondary anti-Mouse-HRP (Southern Biotech, 1031-05) were used where appropriate. Post-HRP imaging, phospho-specific immunoblots are stripped and re-incubated with corresponding total protein antibody sequentially from the same membrane. Antibodies for DNA damage response markers are anti-phospho-CHK1 Ser345 [clone 133D3] (CST, 2348P; RRID:AB_331212) and anti-phospho-H2AX Ser139 [clone 20E3] (CST, 9718P; RRID:AB_2118009). To assay carfilzomib induced caspase cleavage of spliceosome components, MM.1S cells were probed with anti-Caspase3 Asp175 [clone 5A1E] (CST, 9664T; RRID:AB_2070042), anti-U2AF65/U2AF2 (Abcam, ab37483; RRID:AB_883338, diluted 1:250 v/v), anti-SF3A1 [clone EPR7667] (Abcam, ab128898); SF3B1 [clone D7L5T] (CST, 14434S). In mechanistic experiments comparing SRSF10 signaling to heat shock response, immunoblots used anti-SRSF10/FUSIP1 [clone T-18] (SCBT, sc-101132; RRID: AB_1123037), anti-HSP27 [clone G31] (CST, 2402; RRID:AB_331761), and anti-β-actin [clone 8H10D10] (CST, 3700; RRID:AB_2242334). Uncropped blots are provided as a Source Data file.

SRSF1 phosphorylation Immunoblot

For SRSF phosphorylation immunoblot in **Supplementary Fig. 3f**, specific antibodies for phosphorylated SRSF proteins are not commercially available and phosphorylation was determined by gel migration. Cells were gently lysed in 100 mM Tris pH 8.5, 10 mM TCEP (Pierce, 20491), 100 mM NaCl, 1% NP-40 alternative (EMD, 492016), 0.03 U/mL aprotinin (RPI, A20550-0.001) and 1mM PMSF (RPI, P20270-1.0) on ice for 30 min. 20% of MM.1S DMSO sample was set aside and treated with calf intestinal phosphatase (NEB, M0290S) for 1 hr at 37°C in 1X Cutsmart Buffer (NEB, B7204S) to benchmark migration of dephosphorylated species. 1X HALT protease/phosphatase inhibitor was immediately added to all other samples and the remainder of the DMSO sample. The nucleus was separated from lysate by centrifugation and all samples were denatured with 4 M urea (VWR, 97063-802) and 0.1% SDS (Fisher Scientific, AC230420100). Immunoblot with anti-SRSF1 [clone 96] (SCBT, sc-33652; RRID:AB_628248), anti-SRSF3 [clone G-8] (SCBT, sc-398541), and anti-SRSF6 [clone 16H3] (SCBT, sc-57954; RRID:AB_785899). Nuclear fractions were too viscous and ran

with a smear, so were not considered. Uncropped blots are provided as a Source Data file.

RNA-seq library preparation (detailed methods)

Total RNA was extracted with RNeasy Mini-prep kit (Qiagen, 74104). Cells were lysed on ice and genomic DNA was homogenized mechanically using an 18-gauge needle and syringe. For single-timepoint experiments isolated total RNA was cleaned, concentrated with RNA clean & concentrator (Zymo, R1015). mRNA isolation begins with 3 µg total RNA resuspended in lysis/binding buffer, denatured at 65°C for 2 min and treated with SUPERase In (Invitrogen, AM2694), then bound to equilibrated poly-dT magnetic beads (NEB, S1550S), incubated for 10 min, then subjected to a series of washes, according to manufacturer's protocol and eluted with 20 µL RNAse-free water at 80°C for 2-3 min. cDNA library of 200-300 bp fragments was constructed with Illumina platform TruSeq indexed adapters using Hyper Prep RNAseq Illumina kit (Kapa, KK8540), starting with at least 50 ng isolated mRNA. cDNA library between 200-400 bp were isolated by TBE-Urea PAGE (Life Tech, EC68852BOX) stained with SYBR Gold stain (Life Tech, S-11494), imaged on Bio-Rad Chemidoc gel imager, and extracted from the gel by manual excision of bands. The gel pieces are blended by centrifuging through a needle prick hole at the bottom of an eppendorf into a collection tube at maximum speed for 3 min, then heated at 70°C for 10 min in 500 µl 10 mM Tris, pH 8.0 (with occasional vortexing). cDNA that diffused out of the gel matrix was separated from gel particles by spinning through Spin-X concentrator (Corning). cDNA was precipitated with 145 mM NaCl, 15 µg/mL glycogen (Thermo, R0551), 58% isopropanol, incubated in -20°C overnight and centrifuged at 4°C for 45 min. The solution is removed and the precipitated white pellet is gently washed with 900 µl 80% ethanol, centrifuged at maximum speed for 5 min at 4°C. The ethanol is removed and the pellet is dried at room temperature for 10 min, then reconstituted in 5-10 µl 10mM Tris pH 8.0. RNA and DNA quantified at all steps by Nanodrop (Thermo Scientific). cDNA library size and quality were evaluated on a Bioanalyzer 2100 (Agilent) with High Sensitivity DNA Kit (Agilent, 5067-4626), before being submitted for next generation

sequencing on a HiSeq4000 (Illumina) at the UCSF Center for Advanced Technologies laboratory.

For **Supplementary Fig. 7**, RNA-seq libraries for MM.1S cells treated with 5 nM bortezomib for 24 h, or heat shocked at 42C for 4 h, or untreated at 0 hr/baseline, were prepared from poly-A enriched RNA at University of California Davis Genome Center, according to the DNA Tech Core Laboratory procedures for library preparation. These samples were submitted for 150 base pair, paired-end sequencing on a HiSeq4000 at the DNA Tech Core Laboratory, with nearly 40E+6 reads per sample. The sequencing was carried out at the DNA Technologies and Expression Analysis Cores at the UC Davis Genome Center, supported by NIH Shared Instrumentation Grant 1S10OD010786-01.

RNA-seq data analysis

Four libraries from timecourse study were aligned with Bowtie v0.12.8 allowing for up to two mismatches (5). Aligned rRNA and tRNA reads were discarded. Remaining transcripts were aligned to known canonical transcripts of human genome draft GRCh37/hg19. All other libraries were aligned with HISAT (v2.1.0) (6). The mapped reads were converted from sequence alignment map format to binary alignment map format using Samtools (v1.3.1 for timecourse samples and v0.1.19 for all others) (7). Transcriptome assembled and abundance quantified for four libraries from timecourse study by in-house C++ scripts, which assign and count unique reads mapping to canonical hg19 transcripts. Only uniquely mapping reads were used for analysis in **Fig. 1b**. All other binary alignment mapped reads were quantified for gene-level expression with HTSeq (v0.7.2) (8). Differential gene expression analysis for single-timepoint response study was performed in R with DESeq2 and all differential expression lists are deposited in Gene Expression Omnibus (GEO, accession: GSE124510) (9).

Cell fixation and fluorescence imaging

For epifluorescent images in **Fig. 4b**, 5E+5 AMO-1cells expressing SRSF1-WT(-mCherry-NLS-3XFLAG) and SRSF1-SD and SRSF1-SA mutants were harvested from

cell culture, centrifuged at 300 rcf for 5 min. Media was aspirated and the cell pellet was washed once with 1X PBS, centrifuged again for 5 min at 300 rcf and aspirated. The cell pellet was then resuspended in ~200 µl 4% formaldehyde (Pierce, 28906) and incubated in the dark at room temperature for 1h 15 min to fix cells. After fixation, cells were centrifuged at 500 rcf for 5 min, and formaldehyde is carefully removed. Cell pellet was resuspended in PBS and washed twice by centrifugation at 500 rcf for 5 min with aspiration of supernatant. After second wash, cells were resuspended at 5E+6 cells/mL in PBS and pipetted onto poly-L-lysine (Electron Microscopy Sciences, 19320-B) coated #1.5 coverglass (Fisher, 12541B). A small drop of ProLong gold antifade with DAPI (Cell Signaling, 8961S) was added to the cells on the coverslip and gently mixed by stirring with a pipet tip. The coverglass was mounted on glass slide (Fisher, S95933) and cured at room temperature, overnight.

Cells are imaged on a Zeiss Observer Z1 microscope using 63X plan-apochromat oil immersion objective (NA = 1.40). Filter sets for excitation (ex) and emission (em) wavelengths are ex = 335-383 nm, em = 420-470 nm for DAPI and ex = 538-562 nm, em = 570-640 nm, for mCherry. Images were processed in ImageJ (v.1.48) and scale bar represents 10 µm.

For confocal microscopy images in **Supplementary Fig. 8** of AMO-1 expressing SRSF1-mCherry-3XFLAG (SRSF1-noNLS) or control-noNLS (mCherry-3XFLAG), 4E+6 cells/well were plated in a 6-well plate and treated with DMSO or 25 nM cfz for 10 h, the following day. Cells were harvested by centrifugation at 500 rcf for 10 min at room temperature. Pellets were then washed twice with ice-cold PBS followed by cell fixation with 4 % paraformaldehyde (Electron Microscopy Sciences, 15714) in PBS, then deposited on 0.01% Poly-L-ornithine (Sigma, P3655) treated glass bottom dishes (MatTek, P35G-1.5-14-C) for 15 minutes. After fixation, cells were washed twice with PBS, treated with 50 mM ammonium chloride for 15 min, permeabilized with 0.1% Triton/PBS for 15 min, then washed twice with PBS. Next, cells were incubated in blocking solution (5 % BSA in PBS) for an hour at ambient temperature and stained with chicken polyclonal anti-mCherry antibody (dilute 1:1000 with blocking solution, Novus

Biologicals, NBP2-25158; RRID:AB_2636881) overnight at 4°C. The next day, cells were washed thrice with wash buffer (0.1% Triton in PBS), incubated with goat anti-chicken IgG (H+L) conjugated with Alexa 488 (dilute 1:1000 with blocking solution, Invitrogen, A11039; RRID:AB_2534096), and washed five more times with wash buffer. Finally, cells were stained with 2 μg/ml Hoechst33342 DNA stain in PBS (Invitrogen, H1399) and kept in PBS until imaging. Images were acquired on a Leica SP8 confocal microscope using a 63X/NA1.40 objective.

Image analysis was performed using the Fiji package of ImageJ (ver. 2.0.0) software (PMID: 22743772). For each image in **Supplementary Fig. 8a-d**, Z stacks were initially merged to create composite images. All images were then false colored with green for the Hoechst images and red for the mCherry images. In order to analyze the SRSF1-noNLS localization in **Supplementary Fig. 8g**, we first merged two middle slices in Z-series: 24-25 (of 50-60 slices) and subsequently converted the images to 8-bit-grayscale. Images were then analyzed for local thickness at fixed intensity for all images. SRSF1-noNLS showed different localization morphology in the nucleus: 1) total exclusion to nuclear periphery, 2) moderate exclusion, and 3) no exclusion from center of nucleus. Images were then placed in three bins (1, 2 and 3), and at least 50 cells were analyzed for each treatment condition: DMSO or 25 nM cfz for 10 h or heat shock at 42 C for 8 h. **Supplementary Fig. 8h** summarizes the morphology distribution from 2 biological replicates.

E7107 splicing assay

RNA was extracted from AMO-1 and MM.1S with RNeasy Mini-prep kit and 500 ng total RNA was used to reverse transcribe polyA-tail mRNA to cDNA with Verso cDNA synthesis kit (Thermo, AB1453B). Splicing activity was determined by comparing abundance of the mature, spliced form of FBXW5 and MBD4, or the unspliced pre-mRNA of cells between conditions by qPCR with SYBR Green supermix (Bio-Rad, 1725272), in technical triplicate on a StepOne Real-Time PCR system (Applied Biosystems). ΔCt between splice targets and “house-keeping” gene PPIA normalized sample variation, and ΔΔCt reports the fold change difference between E7107 treated

samples in 3 biological replicates and the average of the untreated (DMSO). QPCR primer sequences are in **Supplementary Data 2**.

Patient *ex vivo* flow cytometry data analysis

Flow cytometry data was analyzed with FloJo v.8.8.6 to determine relative abundance of CD138+ cells, with respect to all cells counted. **Supplementary Fig. 11b-c** shows an example dot plot of stained patient samples and **b)** gating strategy for excluding doublets, then debris, and **c)** geometric enclosure used to outline CD138+ cells in both DMSO (0 nM E7107) and 100 nM E7107 samples. Reported percent viability is normalized to amount in DMSO treated samples for each patient. Relative abundance of CD138- cells were counted by excluding CD138+ cells and then considering the relative abundance of live, non-SyTOX green stained cells with respect to all non-CD138+ cells.

SUPPLEMENTARY REFERENCES

1. Yang X, Boehm JS, Yang X, Salehi-Ashtiani K, Hao T, Shen Y*, et al.* A public genome-scale lentiviral expression library of human ORFs. *Nat. Meth.* 2011;8(8):659-61 doi 10.1038/nmeth.1638.

2. Tyanova S, Temu T, and Cox J. The MaxQuant computational platform for mass spectrometry-based shotgun proteomics, *Nat. Prot.* 2016;11:2301-19.

3. Tyanova S, Temu T, Sinitcyn P, Carlson A, Hein M Y, Geiger T, Mann M, and Cox J. The Perseus computational platform for comprehensive analysis of (prote)omics data. *Nat. Meth.* 2016;13:731–740.

4. Wiredja DD, Koyutürk M, Chance MR. The KSEA App: a web-based tool for kinase activity inference from quantitative phosphoproteomics. *Bioinformatics* 2017;33(21):3489-91.

5. Langmead B, Trapnell C, Pop M, Salzberg SL. Ultrafast and memory-efficient alignment of short DNA sequences to the human genome. *Genome Biol* 2009;10:R25 doi.org/10.1186/gb-2009-10-3-r25.

6. Kim D, Langmead B, and Salzberg SL. HISAT: a fast spliced aligner with low memory requirements. *Nat. Meth.* 2015;12(4):357-60.

7. Li H, Handsaker B, Wysoker A, Fennell T, Ruan J, Homer N, *et al*. The Sequence Alignment/Map format and SAMtools. *Bioinformatics* 2009;25(16):2078-9.

8. Anders S and Huber W. Differential expression analysis for sequence count data. *Genome Biol* 2010;11(10):R106 doi 10.1186/gb-2010-11-10-r106.

9. Love MI, Huber W, Anders S. Moderated estimation of fold change and dispersion for RNA-seq data with DESeq2. *Genome Biol* 2014;15(12):550.

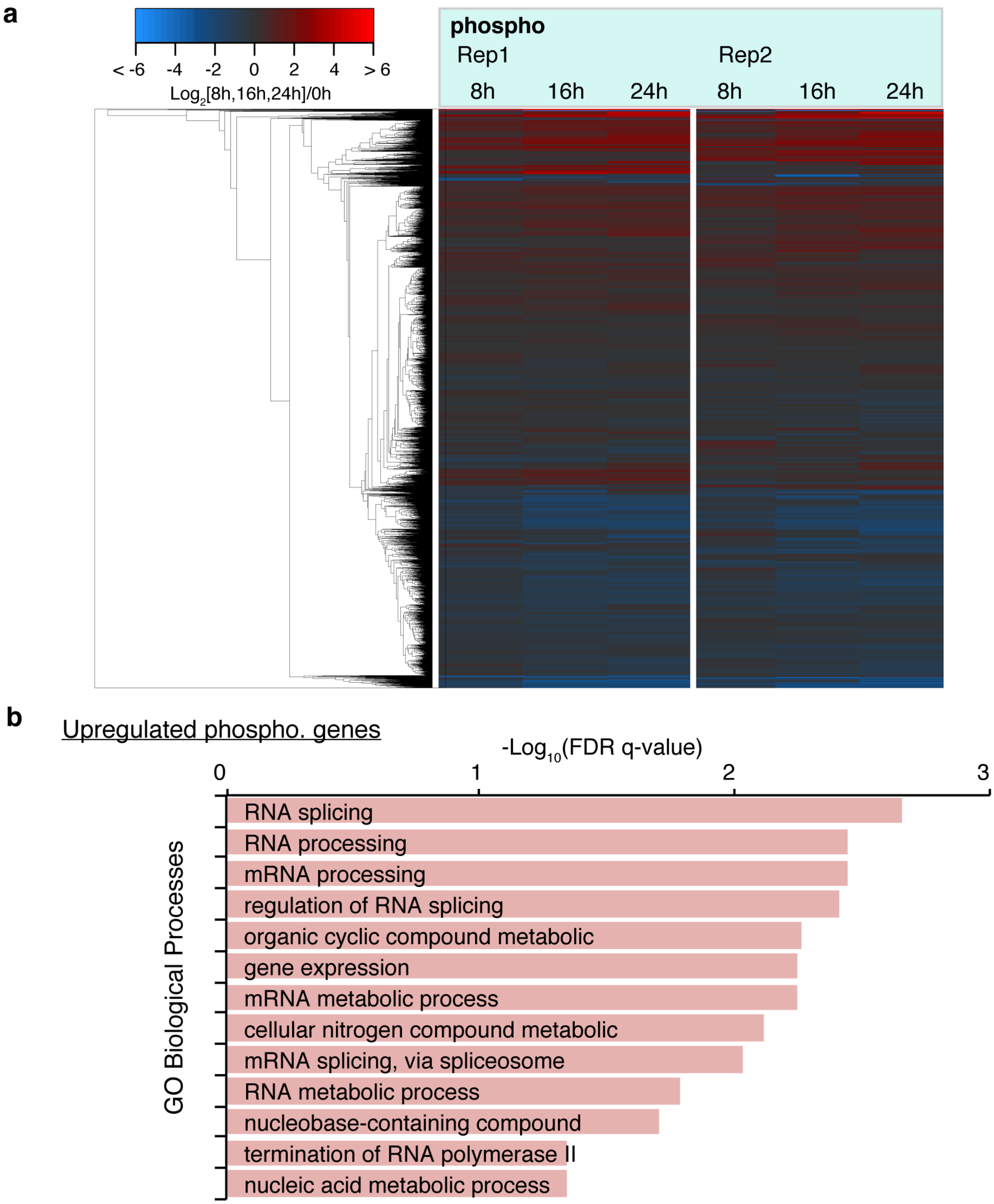

Supplementary Figure 1. Time-course of MM cell phosphorylation after Cfz treatment. a. Heatmap of hierarchical clustering of all 5791 quantified phosphosites, log_2-transformed label-free quantification (LFQ) intensity ratios (relative to 0 hr at 8, 16, 24 hr) for 2 technical replicates of MM.1S cells treated with 30 nM Cfz over a 24 hr time

course. **b.** Top ranked (FDR *q*-value) GO enrichment terms for genes with increased phosphorylation and relatively unchanged transcript levels (**Fig. 1b**) over 24 hr.

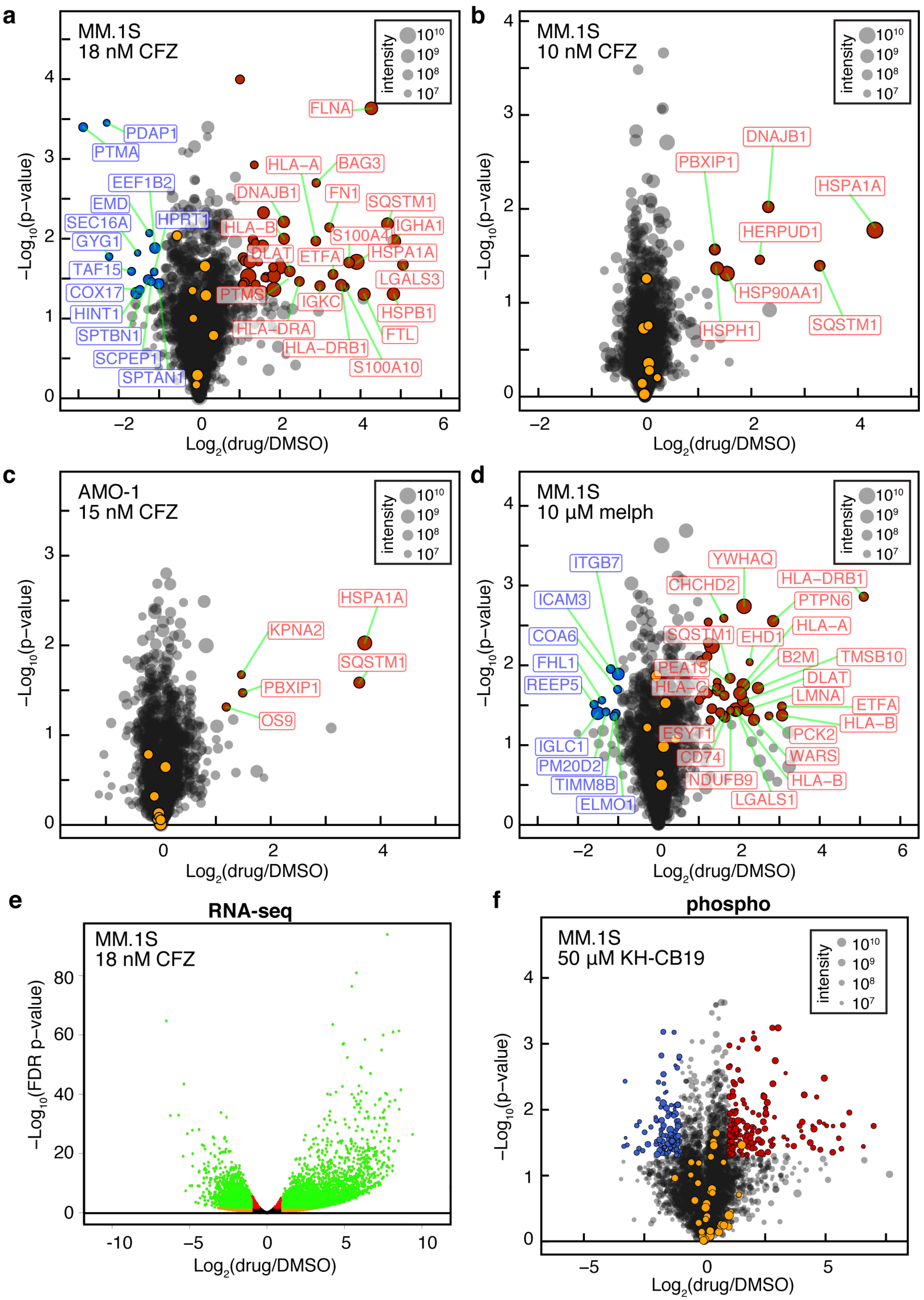
a
MM.1S
18 nM CFZ
b
MM.1S
10 nM CFZ
c
AMO-1
15 nM CFZ
d
MM.1S
10 µM melph
e
RNA-seq
MM.1S
18 nM CFZ
f
phospho
MM.1S
50 µM KH-CB19
intensity
−Log10(p−value)
−Log10(FDR p−value)
Log2(drug/DMSO)
FLNA
PDAP1
PTMA
HLA−A
BAG3
EEF1B2
DNAJB1
FN1
SQSTM1
EMD
HPRT1
SEC16A
HLA−B
S100A4
IGHA1
GYG1
DLAT
ETFA
HSPA1A
TAF15
LGALS3
COX17
PTMS
IGKC
HINT1
HSPB1
SPTBN1
HLA−DRA
FTL
SCPEP1
HLA−DRB1
SPTAN1
S100A10
PBXIP1
HERPUD1
HSP90AA1
HSPH1
KPNA2
OS9
ITGB7
YWHAQ
CHCHD2
HLA−DRB1
ICAM3
PTPN6
COA6
SQSTM1
EHD1
FHL1
PEA15
B2M
TMSB10
REEP5
HLA−C
LMNA
ETFA
IGLC1
ESYT1
PCK2
PM20D2
CD74
WARS
TIMM8B
NDUFB9
ELMO1
LGALS1

Supplementary Figure 2. Protein abundance and gene expression response to drug perturbation. Volcano plots showing $\log_2$ transformed ratios of single time-point SILAC-based LC-MS/MS protein intensities of MM cells treated with **a.** 18 nM Cfz, **b.** 10 nM Cfz. **c.** 15 nM Cfz (AMO-1), or **d.** 10 μM melphalan, compared to DMSO. Red circles are proteins with significantly increased abundances ($p < 0.05$, ≥ 2-fold increase), blue circles are significantly decreased proteins ($p < 0.05$, ≥ 2-fold decrease), and orange circles belong to SRSF family of proteins. Size of dots correspond to summed SILAC light and heavy intensities for each protein. **e.** Volcano plot of $\log_2$ transformed ratio of changes in gene expression for MM.1S treated with 18 nM Cfz, compared to DMSO, with significantly changed genes in green ($p < 0.05$, ≥ 2-fold). **f.** Normalized SILAC LC-MS/MS intensity ratios for phosphopeptides enriched from MM.1S cells treated with 50 μM KH-CB19 versus DMSO for 24 hr. Red and blue circles are significantly changed sites ($p < 0.05$, ≥2-fold). Notably, detected SRSF phosphopeptides (orange circles) do not change significantly in response to CLK1/4 inhibitor. Dot size corresponds to summed SILAC intensities of the phosphopeptides.

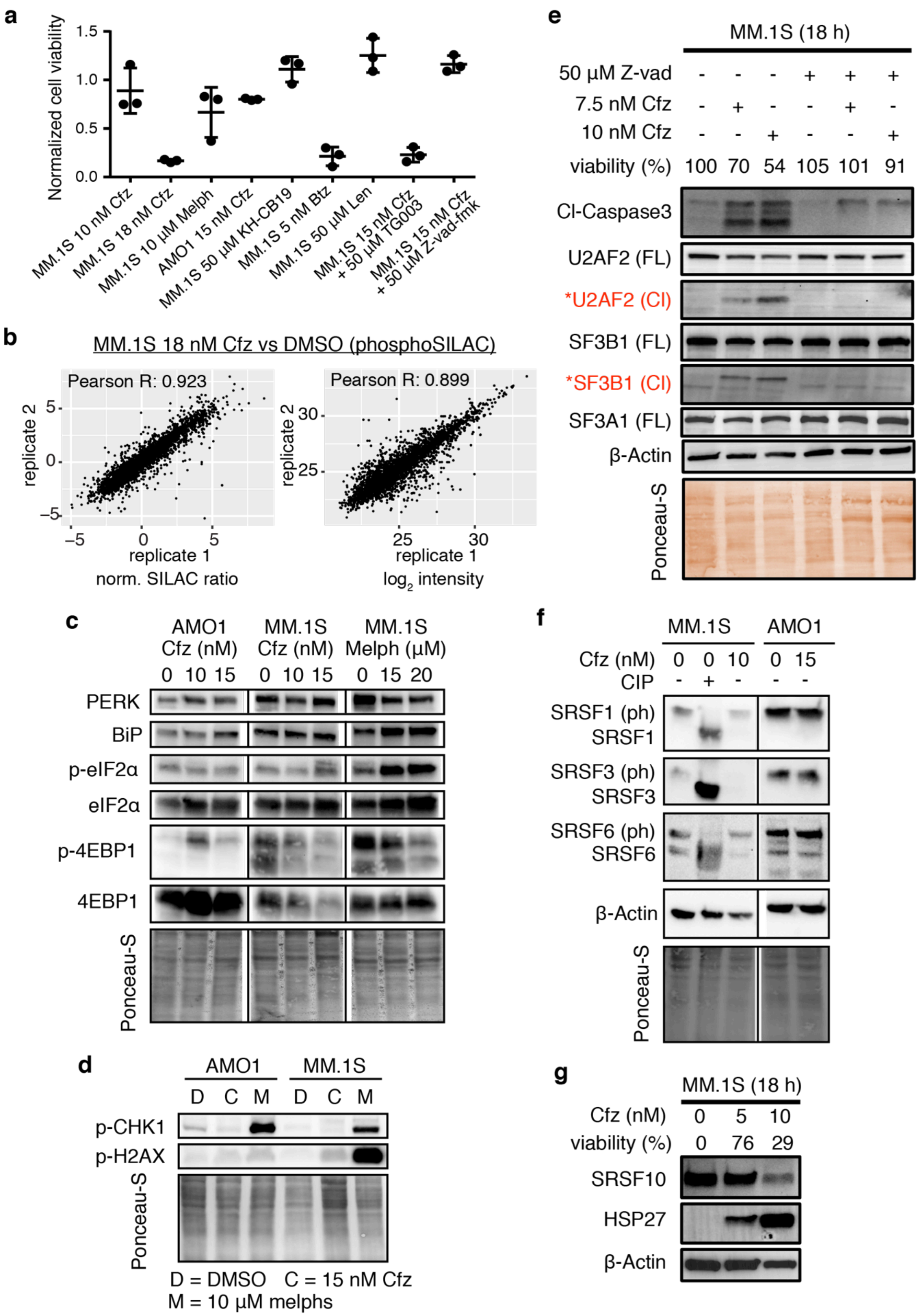
a
Normalized cell viability
1.5
1.0
0.5
0.0
MM.1S 10 nM Cfz
MM.1S 18 nM Cfz
MM.1S 10 µM Melph
AMO1 15 nM Cfz
MM.1S 50 µM KH-CB19
MM.1S 5 nM Btz
MM.1S 50 µM Len
MM.1S 15 nM Cfz + 50 µM TG003
MM.1S 15 nM Cfz + 50 µM Z-vad-fmk
b
MM.1S 18 nM Cfz vs DMSO (phosphoSILAC)
Pearson R: 0.923
replicate 2
replicate 1
norm. SILAC ratio
Pearson R: 0.899
replicate 2
replicate 1
log2 intensity
c
AMO1 Cfz (nM) 0 10 15
MM.1S Cfz (nM) 0 10 15
MM.1S Melph (µM) 0 15 20
PERK
BiP
p-eIF2α
eIF2α
p-4EBP1
4EBP1
Ponceau-S
d
AMO1 D C M
MM.1S D C M
p-CHK1
p-H2AX
Ponceau-S
D = DMSO C = 15 nM Cfz
M = 10 µM melphs
e
MM.1S (18 h)
50 µM Z-vad - - - + + +
7.5 nM Cfz - + - - + -
10 nM Cfz - - + - - +
viability (%) 100 70 54 105 101 91
Cl-Caspase3
U2AF2 (FL)
*U2AF2 (Cl)
SF3B1 (FL)
*SF3B1 (Cl)
SF3A1 (FL)
β-Actin
Ponceau-S
f
MM.1S
AMO1
Cfz (nM) 0 0 10 0 15
CIP - + - - -
SRSF1 (ph)
SRSF1
SRSF3 (ph)
SRSF3
SRSF6 (ph)
SRSF6
β-Actin
Ponceau-S
g
MM.1S (18 h)
Cfz (nM) 0 5 10
viability (%) 0 76 29
SRSF10
HSP27
β-Actin

Supplementary Figure 3. Characterization of myeloma response to Cfz, melphalan, and z-vad-fmk. a. Normalized cell viability (by CellTiter-Glo) vs. DMSO control of MM cells treated with indicated drugs for 24 hr. (n = 3; mean ± S.D.). **b.** Representative scatter plot showing correlation between 2 biological replicates for normalized SILAC ratios (left) and summed (light + heavy) intensities (right) for phosphosites from MM.1S treated with 18 nM Cfz shows high quantitative reproducibility. **c.** Immunoblot of stress biomarkers (PERK, BiP, phospho-/total eIF2α, phospho-/total 4EBP1) for AMO-1 and MM.1S treated with DMSO, 10 nM, 15 nM Cfz, and MM.1S treated with DMSO, 15 μM melphalan, and 20 μM melphalan. Vertical lines indicate excised lanes containing conditions not relevant to this study. **d.** Immunoblot of biomarkers for DNA damage (phospho-CHK1, phospho-H2AX) for AMO-1 and MM.1S treated with DMSO, 15 nM Cfz, and 10 μM melphalan, where activation of DNA damage response occurred much more prominently with melphalan. **e.** Immunoblot of core spliceosome components, U2AF2, SF3B1, and SF3A1 showing Caspase-3 cleavage (* in red) from MM.1S treated with Cfz for 18 hr with and without 50 μM of Caspase inhibitor zVAD-fmk. Cell viability for each condition after 18 hr is included. **f.** Immunoblot of SRSF1, SRSF3, and SRSF6 in cytoplasmic fraction of MM.1S and AMO-1 treated with DMSO and Cfz. Cell extract treated with calf intestinal phosphatase (+ CIP) to highlight mobility shift of phosphorylated species (ph) of SRSF proteins. **g.** Immunoblot of SRSF10 and HSP27 in MM.1S cells treated with DMSO, 5 nM cfz, or 10 nM cfz for 10 h. β-actin immunoblot included to control for load. Cell viability for each condition after 18 h is included. Vertical line between cell lines indicate excised lanes containing conditions not relevant to this study. Ponceau-S stain included as loading control for all immunoblots, except **g**. Source data of uncropped blots are provided as a Source Data file and immunoblots in **S3c, f,** and **g** are representative of results from experiments repeated once and immunoblots in **S3d** and **e** were performed once.

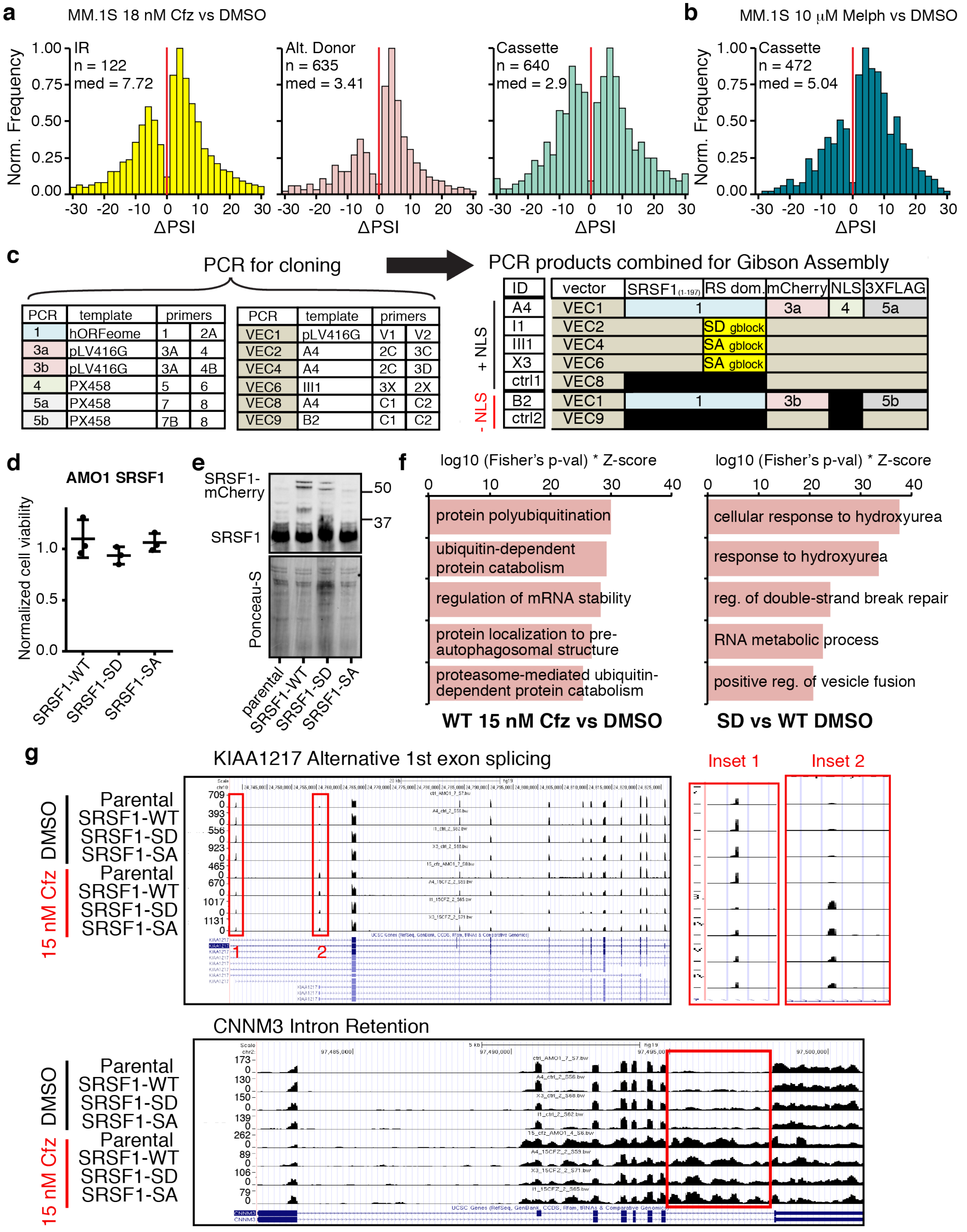
a
MM.1S 18 nM Cfz vs DMSO
Norm. Frequency
IR
n = 122
med = 7.72
Alt. Donor
n = 635
med = 3.41
Cassette
n = 640
med = 2.9
ΔPSI
b
MM.1S 10 μM Melph vs DMSO
Cassette
n = 472
med = 5.04
c
PCR for cloning
PCR products combined for Gibson Assembly
PCR
template
primers
1
hORFeome
1
2A
3a
pLV416G
3A
4
3b
pLV416G
3A
4B
4
PX458
5
6
5a
PX458
7
8
5b
PX458
7B
8
VEC1
pLV416G
V1
V2
VEC2
A4
2C
3C
VEC4
A4
2C
3D
VEC6
III1
3X
2X
VEC8
A4
C1
C2
VEC9
B2
C1
C2
ID
vector
SRSF1(1-197)
RS dom.
mCherry
NLS
3XFLAG
+ NLS
- NLS
A4
I1
III1
X3
ctrl1
B2
ctrl2
SD gblock
SA gblock
d
AMO1 SRSF1
Normalized cell viability
SRSF1-WT
SRSF1-SD
SRSF1-SA
e
SRSF1-mCherry
SRSF1
Ponceau-S
50
37
parental
f
log10 (Fisher's p-val) * Z-score
protein polyubiquitination
ubiquitin-dependent protein catabolism
regulation of mRNA stability
protein localization to pre-autophagosomal structure
proteasome-mediated ubiquitin-dependent protein catabolism
WT 15 nM Cfz vs DMSO
cellular response to hydroxyurea
response to hydroxyurea
reg. of double-strand break repair
RNA metabolic process
positive reg. of vesicle fusion
SD vs WT DMSO
g
KIAA1217 Alternative 1st exon splicing
Inset 1
Inset 2
DMSO
15 nM Cfz
Parental
SRSF1-WT
SRSF1-SD
SRSF1-SA
CNNM3 Intron Retention

Supplementary Figure 4. Cfz-induced splicing alterations across parental MM cells and SRSF constructs. a. and **b.** ΔPSI histograms of significant ($p < 0.05$) IR, alt. exon donor, and alt. cassette splicing events for MM.1S treated with 18 nM Cfz for 24 hr (or **b.** 10 µM melphalan) with respect to DMSO. Subset of data in **Fig. 3b, d**. Bin = 2 and red line at ΔPSI = 0. **c.** List of PCR reactions referencing oligos in **Supplementary Data 2**.and the subsequent Gibson Assemblies to generate lentiviral SRSF1 plasmids. IDs are related to SRSF1-WT and mutants: A4 = WT, I1 = SD, III1/X3 = SA, B2 = WT without NLS. **d.** Normalized cell viability of AMO-1 cells expressing SRSF1-WT, SRSF1-SD, and SRSF1-SA treated with 15 nM Cfz for 24 hr ($n = 3$; mean ± S.D.). **e.** SRSF1 immunoblot of parental AMO-1, AMO-1 expressing SRSF1-WT, SRSF1-SD, and SRSF1-SA, comparing exogenous SRSF1-mCherry-NLS-$[FLAG]_3$ and endogenous SRSF1 abundance. Source data of uncropped blots are provided as a Source Data file and immunoblot was performed once. **f.** Top ranked (combined Fisher's p-value and background weighted Z-score) GO enrichment terms for genes with significant ASE ($p < 0.05$) of all types from WT treated with 15 nM Cfz compared to DMSO (left) and SD compared to WT in DMSO (right). **g.** Examples of Cfz-induced alternative splicing: alternative first exon splicing in *KIAA 1217* (top) and intron retention in *CNNM3* (bottom) for all AMO-1 cells (parental, SRSF1-WT, SD, SA) with 15 nM Cfz compared to DMSO.

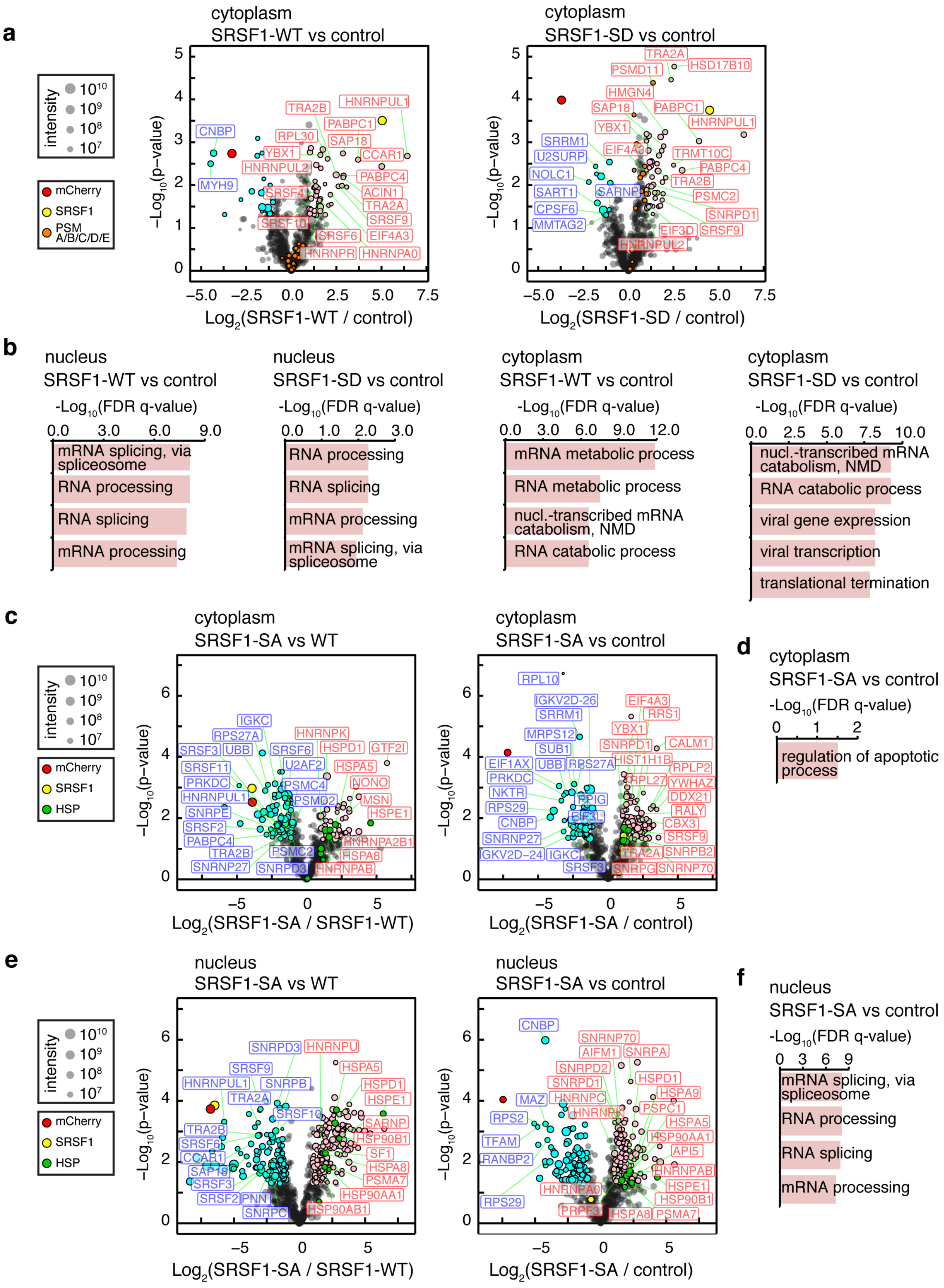
a
cytoplasm
SRSF1-WT vs control
cytoplasm
SRSF1-SD vs control
intensity
10¹⁰
10⁹
10⁸
10⁷
mCherry
SRSF1
PSM A/B/C/D/E
−Log₁₀(p−value)
Log₂(SRSF1-WT / control)
Log₂(SRSF1-SD / control)
b
nucleus
SRSF1-WT vs control
-Log₁₀(FDR q-value)
mRNA splicing, via spliceosome
RNA processing
RNA splicing
mRNA processing
nucleus
SRSF1-SD vs control
-Log₁₀(FDR q-value)
RNA processing
RNA splicing
mRNA processing
mRNA splicing, via spliceosome
cytoplasm
SRSF1-WT vs control
-Log₁₀(FDR q-value)
mRNA metabolic process
RNA metabolic process
nucl.-transcribed mRNA catabolism, NMD
RNA catabolic process
cytoplasm
SRSF1-SD vs control
-Log₁₀(FDR q-value)
nucl.-transcribed mRNA catabolism, NMD
RNA catabolic process
viral gene expression
viral transcription
translational termination
c
cytoplasm
SRSF1-SA vs WT
cytoplasm
SRSF1-SA vs control
intensity
mCherry
SRSF1
HSP
−Log₁₀(p−value)
Log₂(SRSF1-SA / SRSF1-WT)
Log₂(SRSF1-SA / control)
d
cytoplasm
SRSF1-SA vs control
-Log₁₀(FDR q-value)
regulation of apoptotic process
e
nucleus
SRSF1-SA vs WT
nucleus
SRSF1-SA vs control
intensity
mCherry
SRSF1
HSP
−Log₁₀(p−value)
Log₂(SRSF1-SA / SRSF1-WT)
Log₂(SRSF1-SA / control)
f
nucleus
SRSF1-SA vs control
-Log₁₀(FDR q-value)
mRNA splicing, via spliceosome
RNA processing
RNA splicing
mRNA processing

Supplementary Figure 5. The phosphorylation-dependent interactome of SRSF1. **a.** Volcano plot showing AP-MS enriched interaction partners to SRSF1-WT (left panel) and SRSF1-SD (right panel) compared with mCherry-NLS-[FLAG]$_3$ control in AMO-1 cytoplasm. Significantly enriched proteins (p < 0.05, ≥ 2-fold) are in pink and excluded proteins in cyan. Dot size corresponds to combined LFQ intensities for that protein. Proteasomal subunits are colored in orange. **b.** Top ranked (FDR q-value) GO enrichment terms for significantly enriched interaction partners for SRSF1-WT and SRSF1-SD compared to mCherry-NLS-[FLAG]$_3$ control in either the nucleus or cytoplasm of AMO-1. **c and e**. Volcano plots depicting differential interaction partners of SRSF1-SA compared to SRSF1-WT (left panel) and SRSF1-SA enriched proteins compared to control (right panel) in **c)** the cytoplasm and **e)** the nucleus of AMO-1. Significantly enriched and excluded proteins (p < 0.05, ≥ 2-fold) in pink and cyan. Heat shock proteins colored in green. **d and f.** Top ranked (FDR q-value) GO enrichment terms for significantly enriched interaction partners for SRSF1-SA in either the **d)** cytoplasm or **f)** nucleus of AMO-1.

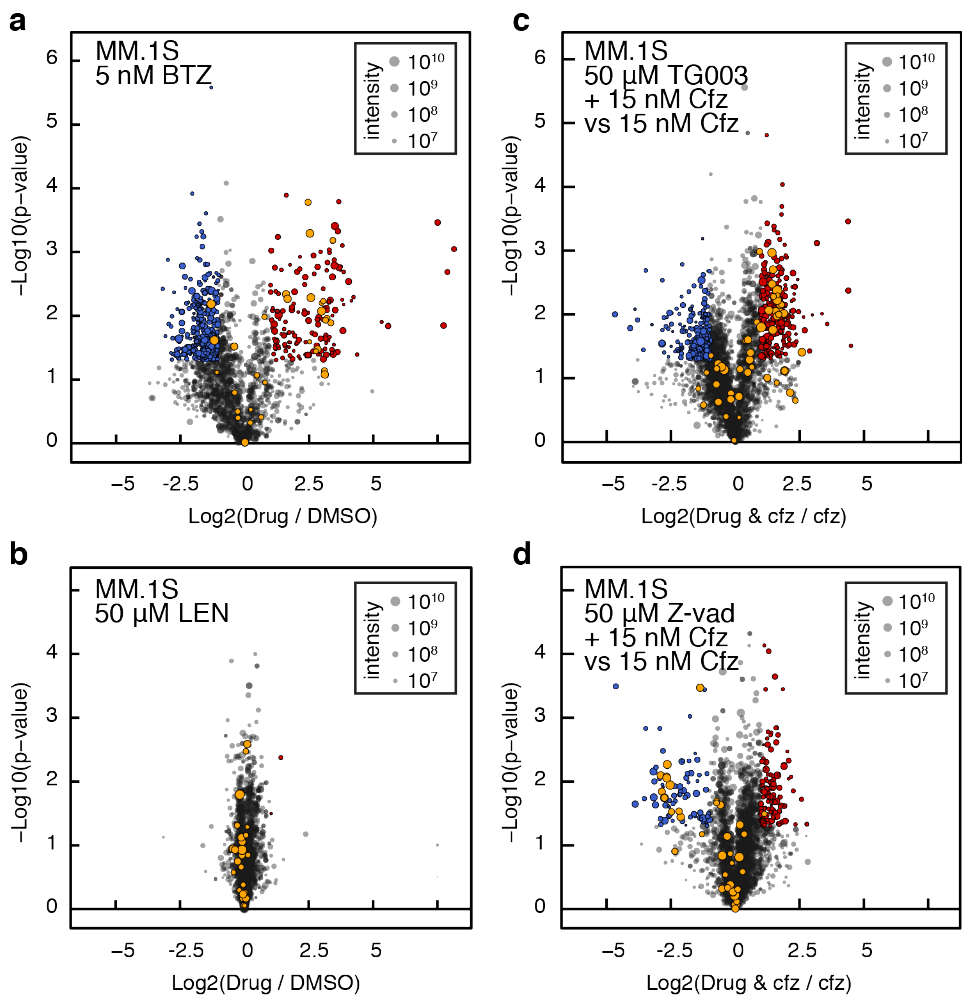

Supplementary Figure 6. Splicing factor phosphorylation is induced in response to btz and blocked by caspase inhibition. a-b. Volcano plots of log_2 transformed ratios of phosphosite abundances between **a.** MM.1S treated with 5 nM bortezomib or **b.** 50 µM lenalidomide compared to DMSO and MM.1S treated with **c.** 15 nM carfilzomib and 50 µM TG003 or **d.** 15 nM carfilzomib and 50 µM z-vad-fmk compared to 15 nM carfilzomib alone. Significant upregulated sites due to drug or drug and cfz are in red, while downregulated are in blue (>2-fold change, $p < 0.05$). SRSF related sites are in orange. Circle size corresponds to summed SILAC intensities.

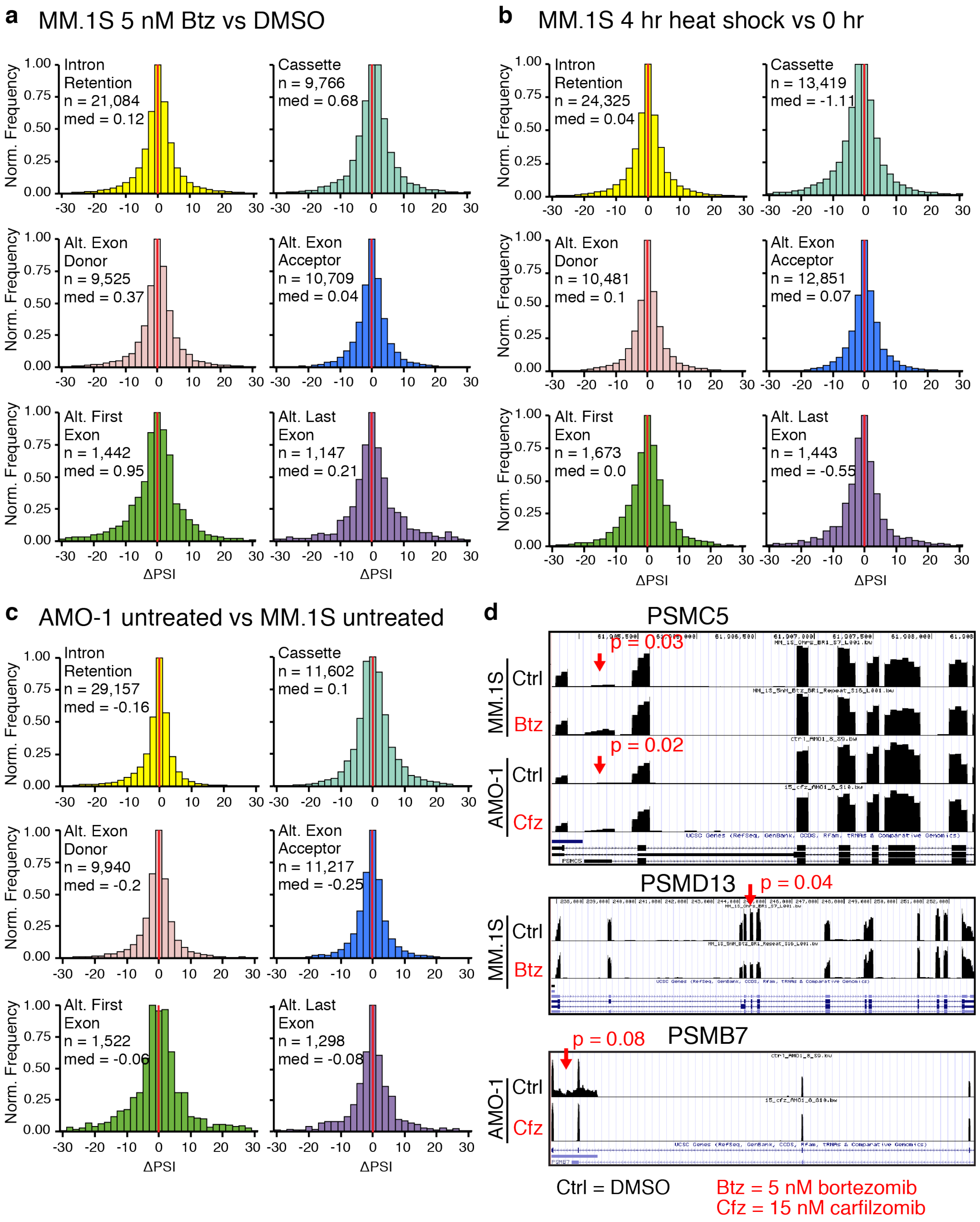

Supplementary Figure 7. Bortezomib and heat shock induce specific splicing, but not global alternative splicing in MM.1S. a-c. Histograms of ΔPSI distributions of

MM.1S treated with 5 nM bortezomib for 24 h compared to untreated control, **b.** MM.1S heat shocked at 42 C for 4 h compared to baseline MM.1S and **c.** DMSO treated MM.1S compared to DMSO treated AMO-1. **d.** RNA-seq coverage map (UCSC Genome Browser) compares counts for the proteasomal subunits *PSMC5* and *PSMD13* between MM.1S treated with 5 nM bortezomib (bottom track) or with DMSO (top track) and compares counts for *PSMC5* (shown in **Fig. 5d**) and *PSMB7* between AMO-1 treated with 15 nm cfz (bottom track) or with DMSO (top track). Red arrow indicates differential splicing and p-value of statistical comparison between treated and untreated samples.

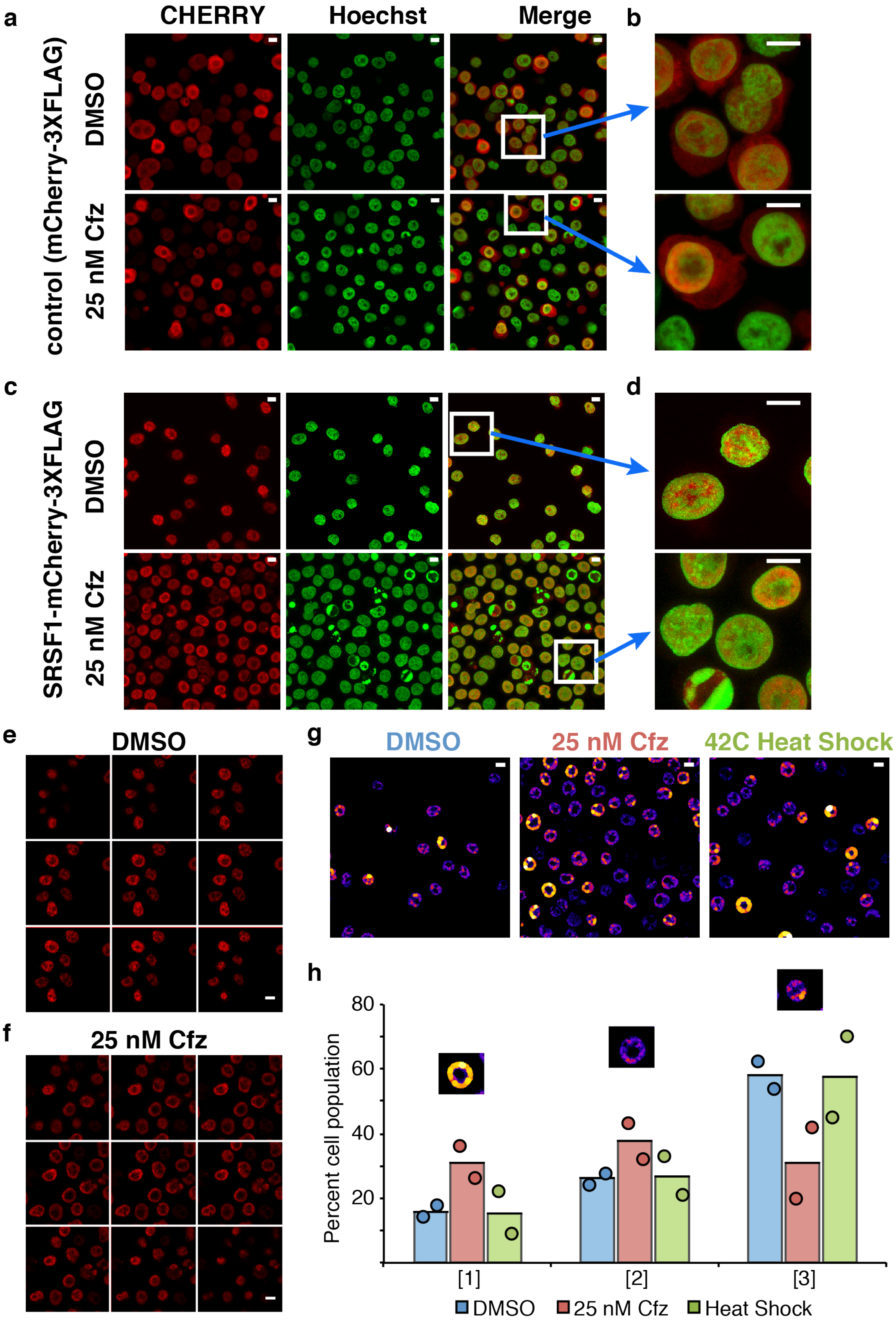

Supplementary Figure 8. Cfz induces SRSF1 localization in the nuclear periphery

of AMO-1. Confocal fluorescence microscopy of AMO-1 expressing either **a.** no NLS control (mCherry-3XFLAG) or **c.** SRSF1-noNLS (SRSF1-mCherry-3XFLAG), treated with either DMSO (top) or 25 nM cfz (bottom) for 10 h. Expanded inset of merged images in **b.** and **d. e-f.** Z-series montage from bottom to top in 1.49 μm increments for SRSF1-noNLS, treated with **e.** DMSO or **f.** 25 nM carfilzomib. **g.** Local thickness analysis of SRSF1-noNLS in AMO-1 treated with DMSO, 25 nM carfilzomib, or heat shock. **a-d, g.** All scale bars in upper right represent 10 μm. **e-f.** All scale bars in lower right represent 10 μm. **h.** Dot plot displaying number of cells out of 100 cells counted for each condition (DMSO, 25 nM carfilzomib, and heat shock) corresponding to one of three SRSF1 localization phenotypes: [1] exclusion to the periphery of the nucleus, [2] moderate exclusion, and [3] not excluded. Bar graphs show mean of n = 2 replicates.

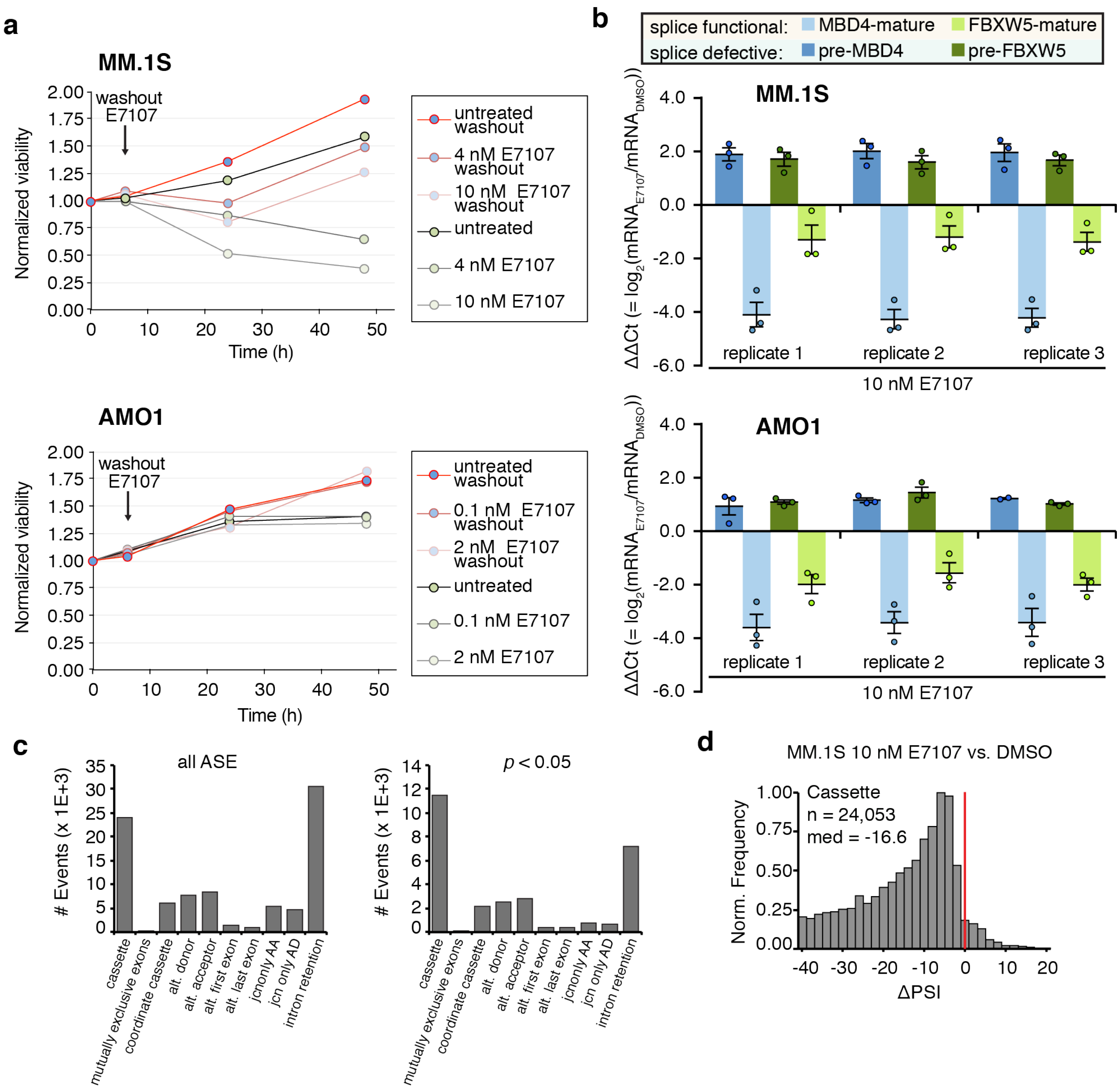

Supplementary Figure 9. E7107 cell toxicity, functional splicing assay, and splicing statistics. a. Normalized cell viability timecourse for MM.1S (top) and AMO-1 (bottom) treated with increasing E7107 concentration. Cells with a washout of E7107 (with exchanged media) at 6 hr have pink and red outlines, while cells without washout are shown with gray and black outlines. **b.** Dot plot depicting n = 3 technical replicates of each log_2 transformed difference of transcript abundance between cells treated with 10 nM E7107 for 6 hr, and DMSO (average of n = 3 measurements), via qPCR ΔΔCt values (normalized to housekeeping gene *PPIA*) of pre-splice and splice competent forms of two targets (*MBD4* in blue and *FBXW5* in green) in MM.1S (top panel) and

AMO-1 cells confirm significant intron retention of canonical targets at this dose and time point. Bar graphs represent mean ± SEM for n = 3 technical replicates. **c.** Distribution of all JuncBASE quantified splice events (n = 89,988) across the ASE types (left panel) and only significant ($p < 0.05$) events (n = 28,436) (right panel) in MM.1S treated with 10 nM E7107 for 6 hr compared to DMSO shows IR and alternative cassette exon splicing events are the most common types, regardless of significance. **d.** ΔPSI histogram of cassette events for MM.1S treated with 10 nM E7107 for 6 hr with respect to DMSO shows the effect of impaired splicing on exon selection. Bin = 2 and red line at ΔPSI = 0.

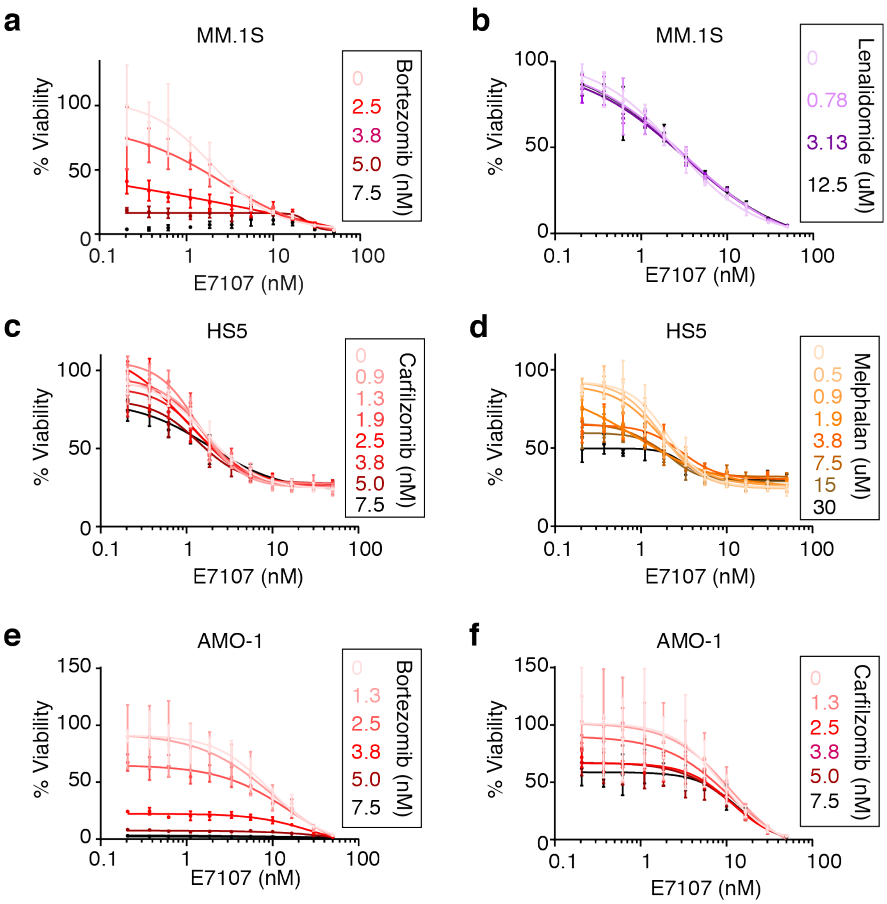

Supplementary Figure 10. **E7107 has specific anti-MM activity in synergy with PI.** **a-b.** Cell viability curves of MM.1S combination therapy with E7107 and **a.** bortezomib or **b.** lenalidomide (n = 4; mean ± S.D.). **c-d.** Viability curves of stromal cell line, HS5, combination therapy with E7107 and **c.** carfilzomib or **d.** melphalan. (n = 4; mean ± S.D.) **e-f.** Viability curves of AMO-1 combination therapy with E7107 and **e.** bortezomib or **f.** carfilzomib (n = 4; mean ± S.D.). ZIP synergy scores are summarized in **Fig. 6i**.

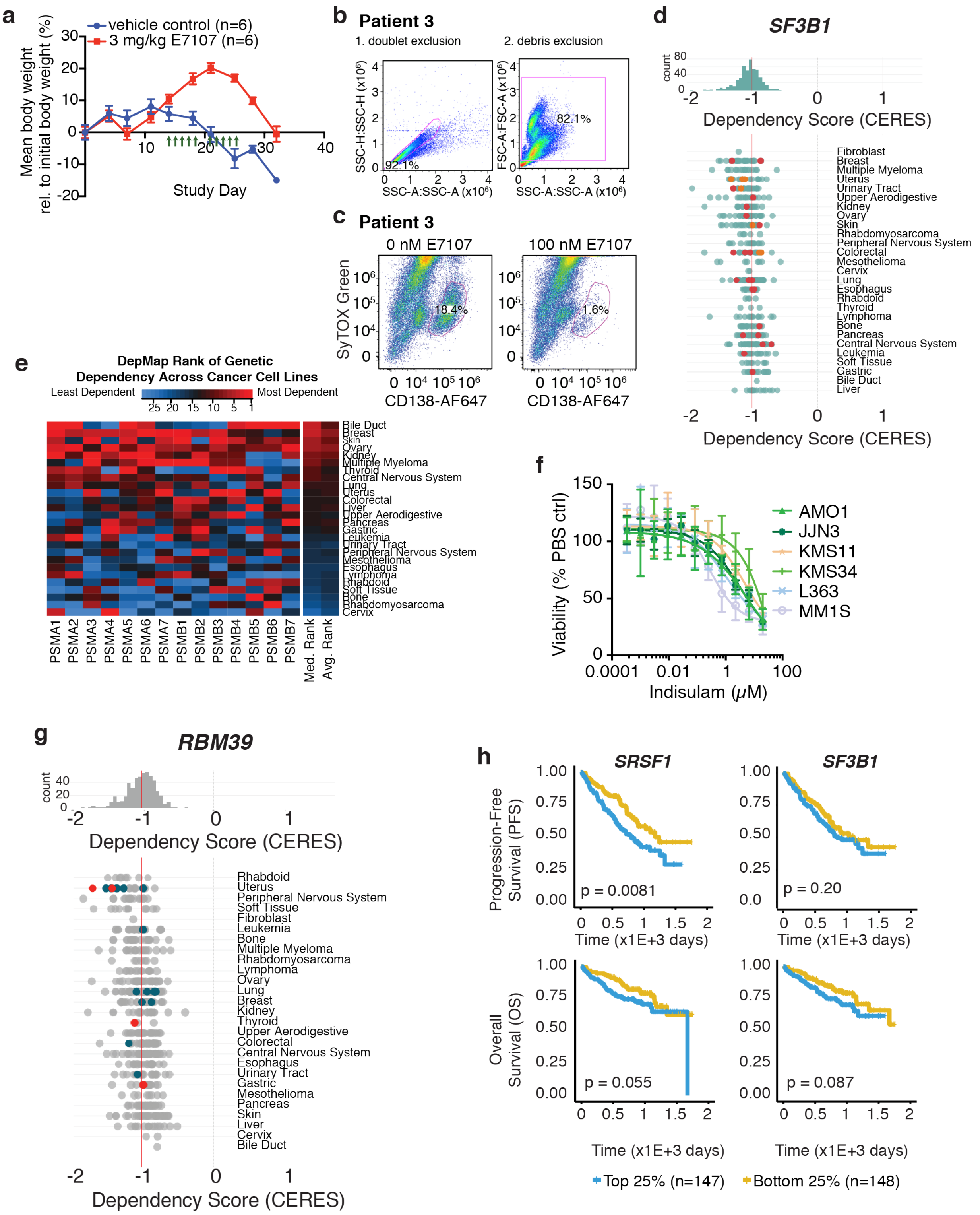

Supplementary Figure 11. Preclinical and clinical relevance of targeting the spliceosome in myeloma. a. 3 mg/kg I.V. E7107 leads to minimal weight loss in NSG mice (mean ± S.D.; n = 6 per arm). **b.** Flow cytometry dot plots demonstrating gating

strategy from primary MM patient bone marrow aspirates, first excludes doublets, then debris. Cells included for downstream analysis are enclosed by pink outlines. **c**. After filtering in **b**., primary plasma cell (CD138+) percentage is then determined amongst live cells (SyTOX-Green). Gated region indicates CD138+ plasma cells. **d.** DepMap (www.depmap.org) CRISPR screen (**Avana** library **18Q4**) dependency data indicating that MM cell lines are among the most sensitive to *SF3B1* depletion based on cell line rank (1 = most sensitive, with lowest average CERES score; 27 = least sensitive, with highest average CERES score). Red line indicates cutoff (CERES score = -1) indicative of an essential gene. **e.** Aggregated DepMap ranking of cancer cell lines across all genes comprising the 20S proteasome, indicating increased but not maximal sensitivity of MM lines to genetic proteasome subunit ablation. **f.** Cytotoxicity of indisulam versus a panel of MM cell lines (48 hr treatment; n = 4 per data point, mean ± S.D.). **g.** DepMap dependency data (as in **e.**) for RBM39. **h.** Progression-free and overall survival data from the CoMMpass study (version **IA11**) with respect to top and bottom quartiles of *SRSF1* and *SF3B1* gene expression in newly-diagnosed MM patient tumor cells. *p*-values by two-sided log-ranked test.

Supplementary Table 1. Genetic constructs. Description of SRSF1 constructs

ID	Name	Features	Promoter	Details
**A4**	SRSF1 WT	SRSF1-mCherry-NLS-3XFLAG	EF1α	human SRSF1 was obtained from hORFeome v8.1; plasmid backbone was amplified by PCR from pLV416G-f-luc/mCherry lentiviral vector used for constitutive expression of luciferase in mouse models
**B2**	SRSF1-noNLS	SRSF1-mCherry-3XFLAG	EF1 α	human SRSF1 was obtained from hORFeome v8.1; plasmid backbone was amplified by PCR from pLV416G-f-luc/mCherry lentiviral vector used for constitutive expression of luciferase in mouse models
**I1**	SRSF1 mSD	SRSF1(1-197)-all RS domain S--> D substitution-mCherry-NLS-3XFLAG	EF1α	templated from A4 construct
**X3**	SRSF1 mSA	SRSF1(1-197)-all RS domain S--> A substitution-mCherry-NLS-3XFLAG	EF1α	templated from III1 construct, which was initial SRSF1mSA construct with shortened mCherry linker
**ctrl1**	ctrl1	mCherry-NLS-3XFLAG	EF1α	templated from A4 construct
