# Supplementary material for: Proteasome inhibitor-induced modulation reveals the spliceosome as a specific therapeutic vulnerability in multiple myeloma
Source: Nat Commun. 2020 Apr 22;11:1931. doi: 10.1038/s41467-020-15521-4 (PMC7176739; doi:10.1038/s41467-020-15521-4)
Supplement: Supplementary file 3 — Description of Additional Supplementary Files [file 41467_2020_15521_MOESM3_ESM.pdf]

Description of Additional Supplementary Files

File Name: Supplementary Data 1
Description: Excel file with tabs listing comparative analysis (T-test *p*-value and log_2-difference) of phospho- and global proteomics

File Name: Supplementary Data 2
Description: Oligo sequences for cloning, qPCR, and RNA-seq cDNA library.

File Name: Supplementary Data 3
Description: Distribution statistics of ΔPSI calculated in R. Statistics for individual event types and all event types are listed for each comparative condition.

File Name: Supplementary Data 4
Description: Excel file with tabs summarizing bioinformatics GO enrichment analysis and KSEA kinase scores.

File Name: Supplementary Data 5
Description: Excel file listing splicing gene mutations and variant allele frequencies found in MM patients from CoMMpass dataset.
