# Supplementary material for: Proteasome inhibitor-induced modulation reveals the spliceosome as a specific therapeutic vulnerability in multiple myeloma
Source: Nat Commun. 2020 Apr 22;11:1931. doi: 10.1038/s41467-020-15521-4 (PMC7176739; doi:10.1038/s41467-020-15521-4)
Supplement: Supplementary file 9 — Reporting Summary [file 41467_2020_15521_MOESM9_ESM.pdf]

nature research

Corresponding author(s): Arun P. Wiita

Last updated by author(s): Dec 24, 2019

Reporting Summary

Nature Research wishes to improve the reproducibility of the work that we publish. This form provides structure for consistency and transparency in reporting. For further information on Nature Research policies, see Authors & Referees and the Editorial Policy Checklist.

Statistics

For all statistical analyses, confirm that the following items are present in the figure legend, table legend, main text, or Methods section.

n/a	Confirmed	
☐	☒	The exact sample size (*n*) for each experimental group/condition, given as a discrete number and unit of measurement
☐	☒	A statement on whether measurements were taken from distinct samples or whether the same sample was measured repeatedly
☐	☒	The statistical test(s) used AND whether they are one- or two-sided<br>*Only common tests should be described solely by name; describe more complex techniques in the Methods section.*
☒	☐	A description of all covariates tested
☐	☒	A description of any assumptions or corrections, such as tests of normality and adjustment for multiple comparisons
☐	☒	A full description of the statistical parameters including central tendency (e.g. means) or other basic estimates (e.g. regression coefficient) AND variation (e.g. standard deviation) or associated estimates of uncertainty (e.g. confidence intervals)
☐	☒	For null hypothesis testing, the test statistic (e.g. *F*, *t*, *r*) with confidence intervals, effect sizes, degrees of freedom and *P* value noted<br>*Give P values as exact values whenever suitable.*
☒	☐	For Bayesian analysis, information on the choice of priors and Markov chain Monte Carlo settings
☒	☐	For hierarchical and complex designs, identification of the appropriate level for tests and full reporting of outcomes
☒	☐	Estimates of effect sizes (e.g. Cohen's *d*, Pearson's *r*), indicating how they were calculated

Our web collection on statistics for biologists contains articles on many of the points above.

Software and code

Policy information about availability of computer code

Data collection	All proteomic samples were analyzed on a Q-Exactive plus MS with commercial software Xcalibur v4.1 (Thermo Scientific).
Data analysis	All mass spec data was searched and quantified in MaxQuant v.1.5.1.2, v1.6.0.16, v1.6.2.1. Downstream analysis and statistical T-tests for phospho-/global/AP-MS proteomics were conducted in Perseus v1.6.2.2. Volcano plots, histograms and histogram statistics were executed in R v3.5.1. Dose-response curves were plotted with GraphPad Prism v6. Flow cytometry data was analyzed with FloJo v8.8.6. RNA-seq data was aligned with Bowtie v0.12.8 or HiSat2 v2.1.0 and binary map formatting with Samtools v1.3.1 and v.0.1.19. Sequencing reads were quantified with HTSeq v0.7.2 and differential expression was analyzed in DESeq2. JuncBASE v1.2 was used to analyze alternative splicing. ImageJ v.1.48 was used to crop and false-color/merge epi-fluorescent images and ImageJ v 2.0.0 in the Fiji package was used for confocal fluorescence stack analysis and Living Image Software (PerkinElmer) was used to quantify Bio-luminescent imaging of mouse tumors. Web application SynergyFinder (https://synergyfinder.fimm.fi/synergy/20200223025730555027/)

For manuscripts utilizing custom algorithms or software that are central to the research but not yet described in published literature, software must be made available to editors/reviewers. We strongly encourage code deposition in a community repository (e.g. GitHub). See the Nature Research guidelines for submitting code & software for further information.

Data

Policy information about availability of data

All manuscripts must include a data availability statement. This statement should provide the following information, where applicable:

- Accession codes, unique identifiers, or web links for publicly available datasets
- A list of figures that have associated raw data
- A description of any restrictions on data availability

The mass spectrometry proteomics data and MaxQuant analysis results have been deposited to the ProteomeXchange Consortium via the PRIDE (58) partner repository with the dataset identifier PXD012172.

Raw RNA-seq data, processed analysis files, and JuncBASE results may be downloaded from the Gene Expression Omnibus, GEO (https://www.ncbi.nlm.nih.gov/geo/) with the accession number: GSE124510.

Field-specific reporting

Please select the one below that is the best fit for your research. If you are not sure, read the appropriate sections before making your selection.

☒ Life sciences ☐ Behavioural & social sciences ☐ Ecological, evolutionary & environmental sciences

For a reference copy of the document with all sections, see nature.com/documents/nr-reporting-summary-flat.pdf

Life sciences study design

All studies must disclose on these points even when the disclosure is negative.

Sample size	No sample size calculation was performed. Sample sizes (number of biological replicates) were determined according to standards in the field (for example, PMID: 29359686, 31182586, 29535314).
Data exclusions	No data was excluded
Replication	All proteomics and RNA-seq analyses were conducted on 2-3 biological replicates. For Proteomic samples, Pearson correlation of log2 Intensity was performed between replicates to ensure reproducibility between samples such as in supplementary figure S3B. Pearson correlation R >0.6 for all comparisons between replicates. Western blots (except for Supplementary Figure 3f and Supplementary Figure 4e) were repeated at least once with similar results, but only one blot is included in analysis. Cell viability experiments were performed with 4 technical replicate measurements, but were repeated once with similar results. Mice experiments were performed with 6 mice per arm and the standard deviation in tumor burden in Fig. 7b shows very tight distribution within each group.
Randomization	MM.1S injected mice were randomized for drug or vehicle selection such that each cohort had approximately same tumor distribution on day of drug application. Proteomic experiments were not randomized because sample batches were small, but blanks were injected in between sample injections to minimize carryover. All ex vivo CD138+ plasma samples from MM patients were similarly treated with and without E7107 drug. Additional randomization was not performed as all other experiments were performed simultaneously with controls on identical cell populations.
Blinding	Blinding was not routinely performed in this study due to necessity of maintaining sample labeling for combined replicate analysis between groups for proteomic and RNA-seq data. Blinding also is not technically feasible in our drug screening or primary patient sample assays given the setup of the plate-based, medium-throughput experimental assay.

Reporting for specific materials, systems and methods

We require information from authors about some types of materials, experimental systems and methods used in many studies. Here, indicate whether each material, system or method listed is relevant to your study. If you are not sure if a list item applies to your research, read the appropriate section before selecting a response.

Materials & experimental systems

n/a	Involved in the study	
☐	☒	Antibodies
☐	☒	Eukaryotic cell lines
☒	☐	Palaeontology
☐	☒	Animals and other organisms
☐	☒	Human research participants
☒	☐	Clinical data

Methods

n/a	Involved in the study	
☒	☐	ChIP-seq
☐	☒	Flow cytometry
☒	☐	MRI-based neuroimaging

Antibodies

Antibodies used	Primary antibodies for probing stress response initiation are anti-PERK [clone D11A8] (CST, 5683P; RRID:AB_10841299), anti-BiP polyclonal (CST, 3183S; RRID:AB_10695864), anti-phospho-eIF2α Ser51 [clone D9G8] (CST, 3398P; RRID:AB_2096481), anti-eIF2α [clone D7D3] (CST, 5324S; RRID:AB_10692650), anti-phospho-4EBP1 Thr37/46 [clone 236B4] (CST, 2855S; RRID:AB_560835), and anti-4EBP1 [clone 53H11] (CST, 9644P; RRID:AB_2097841)<br>Antibodies for DNA damage response markers are anti-phospho-CHK1 Ser345 [clone 133D3] (CST, 2348P; RRID:AB_331212) and anti-phospho-H2AX Ser139 [clone 20E3] (CST, 9718P; RRID:AB_2118009).<br>Antibodies for caspase activity on splice factors are anti-Caspase3 Asp175 [clone 5A1E] (CST, 9664T; RRID:AB_2070042), anti-U2AF65=U2AF2 polyclonal (Abcam, ab37483; RRID:AB_883338), anti-SF3A1 [clone EPR7667] (Abcam, ab128898), anti-SF3B1 [clone D7L5T] (CST, 14434S)<br>SRSF proteins were detected by immunoblots with anti-SRSF1 [clone 96] (SCBT, sc-33652; RRID:AB_628248), anti-SRSF3 [clone G-8] (SCBT, sc-398541), and anti-SRSF6 [clone 16H3] (SCBT, sc-57954; RRID:AB_785899).<br>Horseradish Peroxidase conjugated antibodies: anti-beta-actin-HRP [clone 13E5] (CST, 5125S; RRID:AB_1903890), secondary

anti-Rabbit F(ab)-HRP (Southern Biotech, 4052-05) and 1:3000 secondary anti-Mouse-HRP (Southern Biotech, 1031-05) were used to detect proteins by immunoblots.
For flow cytometry, patient cells were stained with Alexa-Fluor 647 mouse anti-human CD138 antibody [Clone MI15] (BD Pharmingen, cat# 562097; RRID:AB_10895974) or Alexa-Fluor 647 IgG κ isotype [clone MOPC-21] (BD Pharmingen, cat# 557714; RRID:AB_396823) control antibody.
Antibodies for SRFS10 and Heat shock: anti-SRSF10=FUSIP1 [clone T-18] (SCBT, sc-101132; RRID: AB_1123037), anti-HSP27 [clone G31) (CST, 2402T; RRID:AB_331761), and anti-β-actin [clone 8H10D10] (CST, 3700T; RRID:AB_2242334)
Antibodies used for confocal imaging: chicken polyclonal anti-mCherry (Novus Biologicals, NBP2-25158; RRID:AB_2636881) and goat anti-chicken IgG (H+L) conjugated with Alexa 488 (Invitrogen, A11039; AB_2534096)
(CST = Cell Signaling Technology, SCBT = Santa Cruz Biotechnology)

Validation

All antibodies produced bands at the desired molecular weight and have been previously validated by the manufacturer: PERK (https://media.cellsignal.com/pdf/5683.pdf), BiP (https://media.cellsignal.com/pdf/3183.pdf), phospho-eIF2α (https://media.cellsignal.com/pdf/3398.pdf), eIF2α [clone D7D3) (https://media.cellsignal.com/pdf/5324.pdf), phospho-4EBP1 (https://media.cellsignal.com/pdf/2855.pdf), and 4EBP1 (https://media.cellsignal.com/pdf/9644.pdf), phospho-CHK1 (https://media.cellsignal.com/pdf/2348.pdf), and phospho-H2AX (https://media.cellsignal.com/pdf/9718.pdf), Caspase3 (https://media.cellsignal.com/pdf/9664.pdf), U2AF65=U2AF2 (archived data sheet: https://www.abcam.com/u2af65-antibody-ab37483.html), SF3A1(archived data sheet: https://www.abcam.com/sf3a1-antibody-epr7667-ab128898.html), SF3B1 (https://media.cellsignal.com/pdf/14434.pdf),SRSF1 (https://datasheets.scbt.com/sc-33652.pdf), SRSF3 (https://www.scbt.com/p/srp20-antibody-g-8), and SRSF6 (https://www.scbt.com/p/srp55-antibody-16h3), beta-actin-HRP (https://media.cellsignal.com/pdf/5125.pdf), secondary Rabbit F(ab)-HRP (https://www.southernbiotech.com/techbul/4052.pdf)
and secondary Mouse-HRP (https://www.southernbiotech.com/techbul/1031.pdf), Alexa-Fluor 647 mouse human CD138 (https://www.bdbiosciences.com/eu/applications/research/stem-cell-research/hematopoietic-stem-cell-markers/human/negative-markers/alexa-fluor-647-mouse-human-cd138-mi15/p/562097), Alexa-Fluor 647 IgG κ isotype (https://www.bdbiosciences.com/eu/reagents/research/antibodies-buffers/immunology-reagents/human-antibodies/cell-surface-antigens/alexa-fluor-647-mouse-igg1-isotype-control-mopc-21/p/557714), SRSF10 (https://www.scbt.com/p/fusip1-antibody-t-18), HSP27 (https://media.cellsignal.com/pdf/2402.pdf), and β-actin [clone 8H10D10] (https://media.cellsignal.com/pdf/3700.pdf), polyclonal mCherry (https://www.novusbio.com/products/mcherry-antibody_nbp2-25158), and IgG (H+L) conjugated with Alexa 488 (https://www.thermofisher.com/antibody/product/Goat-Chicken-IgY-H-L-Secondary-Antibody-Polyclonal/A-11039)

Eukaryotic cell lines

Policy information about cell lines

Cell line source(s)

MM.1S is from ATCC (CRL-2974); L363, RPMI8266, JJN3 are from Deutsche Sammlung von Mikroorganismen und Zellkulturenrepository (DSMZ; ACC 49, ACC 402, ACC 541 respectively); AMO-1 was originally from DSMZ (ACC 538) and derived to be Btz-resistant by Dr. Christoph Driessen (Kantonsspital St Gallen); INA6 is a gift courtesy of Dr. Renate Burger (Christian-Albrechts-Universität zu Kiel), who developed this cell line from a primary myeloma patient sample; KMS34 is from Japanese Collection of Research Bioresources Cell Bank (JCRB1195); and MM.1S mCherry/f-luc was provided by UCSF Hematologic Malignancies Tissue Bank (HMTB) and derived from the ATCC stock.

Authentication

All cell lines were validated using STR profiling service by ATCC.

Mycoplasma contamination

No cell lines were positive for mycoplasma.

Commonly misidentified lines
(See ICLAC register)

We did not use any commonly misidentified cell lines

Animals and other organisms

Policy information about studies involving animals; ARRIVE guidelines recommended for reporting animal research

Laboratory animals

NOD.Cg-Prkdcscid Il2rgtm1Wjl/SzJ (NSG) mice were from The Jackson Laboratory (cat# 005557). All the mice were female, 6-8 wks old at start of studies, and typically weigh 20-25 g. NSG mice were handled with aseptic techniques and housed in pathogen free environments at the UCSF Laboratory Animal Research Center.

Wild animals

The study did not involve wild animals.

Field-collected samples

The study did not involve samples collected from the field.

Ethics oversight

All mouse studies were performed according to UCSF Institutional Animal Care and Use Committee-approved protocols.

Note that full information on the approval of the study protocol must also be provided in the manuscript.

Human research participants

Policy information about studies involving human research participants

Population characteristics

De-identified patient samples were from UCSF Hematologic Malignancies Tissue Bank. At the time of collection, samples were from PI-refractory multiple myeloma patients.

Recruitment	Patients were not specifically recruited for this study. All myeloma patients receiving care at UCSF and meeting appropriate inclusion criteria are offered enrollment into the Tissue Bank protocol.
Ethics oversight	Samples were obtained according to UCSF Committee on Human Research-approved protocols and the Declaration of Helsinki

Note that full information on the approval of the study protocol must also be provided in the manuscript.

Flow Cytometry

Plots

Confirm that:

☒ The axis labels state the marker and fluorochrome used (e.g. CD4-FITC).

☒ The axis scales are clearly visible. Include numbers along axes only for bottom left plot of group (a 'group' is an analysis of identical markers).

☒ All plots are contour plots with outliers or pseudocolor plots.

☒ A numerical value for number of cells or percentage (with statistics) is provided.

Methodology

Sample preparation	Mononuclear cells were resuspended in a small volume (~1.5 mL) of media (RPMI1640, 10% FBS, 1% penicillin/streptomycin, 2 mM glutamine) and incubated at 37°C, 5% CO2 for 15 min. Isolated mononuclear cells from multiple myeloma patient bone marrow were adjusted to 2E5 cells/well in a 96 well plate. Cells were stimulated with 50 ng/ml recombinant human IL-6 (ProsPec) for 17 hr before treatment with E7107 or DMSO for 24 hr (or 40 hr in Patient 4 sample). Cells were then stained with 10 µL Alexa-Fluor 647 mouse anti-human CD138 antibody (BD Pharmingen, cat# 562097; RRID:AB_10895974) or Alexa-Fluor 647 IgG κ isotype (BD Pharmingen, cat# 557714; RRID:AB_396823) control and 2 µL SyTOX Green (Thermo, S34860) per 1 mL FACS buffer (D-PBS, 5% FBS).
Instrument	Cells were characterized with a CytoFLEX fluorescence cytometer (BD).
Software	Flow cytometry data was analyzed with FloJo v8.8.6.
Cell population abundance	CD138 positive cell abundance were 1.6%, 25.8%, 20.7%, 10.7%, 2.6%, 0.34%, 0.82% of the total flow analyzed sample for patients 1-7, respectively.
Gating strategy	Samples were gated first for cell doublet exclusion by SSC-A vs SSC-H, then cell debris exclusion by excluding bottom left corner of FSC-A vs SSC-A plots (as shown in supplementary figure S7b). Gating for CD138 positive cell population was chosen individually for each patient sample and polygon selection of CD138 positive cells in DMSO treated samples were bimodally distributed from CD138 negative cells in CD138-AF647 vs SYTOX-green plots (as shown in supplementary figure S7c). Isotype control and unstained samples confirmed specificity of gates.

☒ Tick this box to confirm that a figure exemplifying the gating strategy is provided in the Supplementary Information.
